# Rampant Exchange of the Structure and Function of Extramembrane Domains between Membrane and Water Soluble Proteins

**DOI:** 10.1371/journal.pcbi.1002997

**Published:** 2013-03-21

**Authors:** Hyun-Jun Nam, Seong Kyu Han, James U. Bowie, Sanguk Kim

**Affiliations:** 1 School of Interdisciplinary Bioscience and Bioengineering, Department of Life Science, Division of IT Convergence Engineering, Pohang University of Science and Technology, Pohang, Korea; 2 Department of Chemistry and Biochemistry, UCLA-DOE Institute of Genomics and Proteomics, Molecular Biology Institute, University of California Los Angeles, Los Angeles, California, United States of America; Stockholm University, Sweden

## Abstract

Of the membrane proteins of known structure, we found that a remarkable 67% of the water soluble domains are structurally similar to water soluble proteins of known structure. Moreover, 41% of known water soluble protein structures share a domain with an already known membrane protein structure. We also found that functional residues are frequently conserved between extramembrane domains of membrane and soluble proteins that share structural similarity. These results suggest membrane and soluble proteins readily exchange domains and their attendant functionalities. The exchanges between membrane and soluble proteins are particularly frequent in eukaryotes, indicating that this is an important mechanism for increasing functional complexity. The high level of structural overlap between the two classes of proteins provides an opportunity to employ the extensive information on soluble proteins to illuminate membrane protein structure and function, for which much less is known. To this end, we employed structure guided sequence alignment to elucidate the functions of membrane proteins in the human genome. Our results bridge the gap of fold space between membrane and water soluble proteins and provide a resource for the prediction of membrane protein function. A database of predicted structural and functional relationships for proteins in the human genome is provided at sbi.postech.ac.kr/emdmp.

## Author Summary

Membrane proteins play important roles in cellular communication and molecular transport. However, experimental difficulties and lack of structural information have limited the functional characterization of membrane proteins. In this study, we find that over 60% of the extramembrane domains were structurally related to proteins of known structure. The exchanges between membrane and soluble proteins are particularly frequent in eukaryotes, indicating that this is an important mechanism for increasing functional complexity. This result has important implications for the evolution of membrane and soluble proteins. Beyond that, it provides a previously untapped resource for predicting the functions of many membrane proteins without a known function. Based on these results, we provide a new database of predicted functional and structural overlaps for all membrane proteins in the human genome.

## Introduction

The structural space of soluble proteins has been extensively explored. Indeed, most single-domain soluble proteins now appear to have at least one structural homolog in the current PDB database [1], [2]. In contrast, the exploration of membrane protein fold space lags far behind [3]–[5]. Moreover, much more work has been directed at soluble proteins, so functional annotations are much more extensive for soluble proteins as well.

Membrane proteins reside in a hydrophobic lipid-bilayer, but their extra-membrane regions are exposed to same folding environment as soluble proteins [5]. Thus, fold space of membrane proteins may be connected with soluble proteins through the extra-membrane portions. Indeed, many membrane proteins contain large extracellular domains that can be separated from the membrane embedded domain and they behave as stable soluble proteins. We therefore examined how much overlap exists between the structure spaces of soluble proteins and membrane proteins. If there is extensive domain sharing, it may be possible to use the vast data on soluble proteins to provide information on their membrane protein relatives.

Here, we used a large-scale structure comparison to explore domain sharing between membrane and soluble proteins. We found that: *(i)* a large fraction of membrane proteins share structural similarities with soluble proteins, *(ii)* the domain exchanges between membrane and soluble proteins are particularly frequent in eukaryotes, *(iii)* in many cases, residues in functional sites are conserved between membrane and soluble protein pairs. These results imply that we can use the extensive knowledge of soluble protein function, to infer previously uncharacterized membrane protein functions. We therefore employed structure guided sequence alignment to elucidate the functions of membrane proteins in the human proteome.

## Results

### The fold space of membrane and soluble proteins is highly connected

We compared the structures of the extramembrane domains of 558 membrane proteins with 43,547 soluble protein structure in the PDB by using TM-align [6] which is a suitable tool for large-scale structural comparisons. We found that structure comparison results from various tools were similar (Figure S1A and S1B), but TM-align was faster than other structure alignment programs. Domain structures were considered to be similar if the RMSD was less than 5 Å over an aligned length of more than 100 residues, and a confidence score of more than 0.5 [6].

**Figure 1:**
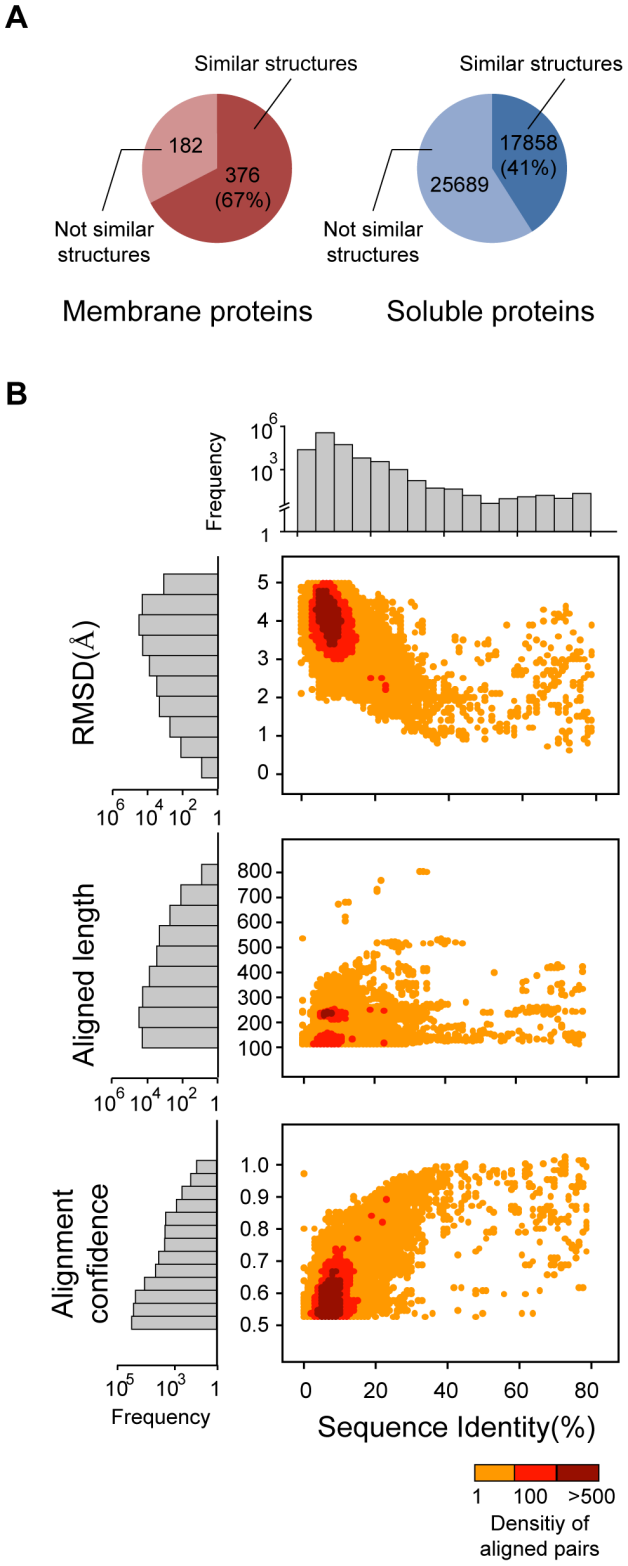
Analyses of the structural alignments between membrane and soluble proteins. (A) Fraction of structurally similar pairs of membrane and soluble proteins. (B) Distribution of RMSD, aligned length, and alignment confidence score according to sequence identities between membrane and soluble proteins.

In the current PDB library, 67% (376) of the membrane proteins share a domain structure with soluble proteins (Figure 1A). Moreover, 41% (17,858) of soluble proteins share structural similarity with the already known membrane protein structures. The structurally similar membrane and soluble proteins have a mean RMSD of 3.9 Å and a mean aligned length of 162 residues. Furthermore, we found that a large fraction of non-redundant membrane protein structures shared extramembrane domains with soluble proteins. We applied PISCES [7] with sequence identity threshold 30% to remove the redundant sequences. Among the 160 non-redundant membrane protein structures, 68% (106) of membrane proteins share extramembrane domains with soluble proteins (Figure S2).

As shown in Figure 1B, the distribution of structural relatives is skewed toward distant relationships with low sequence identity. Thus, most of these relationships would have been undetectable by sequence methods alone, which explains why the high degree of overlap between membrane and soluble protein structures has not been previously observed to our knowledge. The structure alignment data between membrane and soluble proteins are available at: sbi.postech.ac.kr/emdmp. In the web-server, users can search membrane and soluble proteins by PDB ids or Pfam domains and download all structure alignment results (Figure S3).

We found that majority of globular domains shared between membrane and soluble proteins are located at the ‘outside’ region of membrane proteins. We mapped the topology information (i.e. inside and outside regions) onto membrane protein structures aligned with soluble proteins. Among the 376 membrane protein structures, we found that 95.7% (360) structures are located at the ‘outside’ region, whereas only 4.3% (16) structures are located at the ‘inside’ region, suggesting that domain exchange were much more frequent at the outside region of membrane proteins. Interestingly, structures located at the outside region of membrane proteins had larger alignment than structures of inside region. Shared domains located at the outside region have a mean aligned length of 163 residues, whereas domains located at the inside region have a mean aligned length of 116 residues (Figure S4).

The extramembrane domains that have soluble counterpart appear to be less intimately associated with the membrane or membrane embedded domains. To assess the degree of membrane association, we determined the average membrane distance of extramembrane domain, measured by the z-coordinate information from PDBTM database [8] (detailed description in Material and Methods section). The average membrane distance of extramembrane domains that have soluble counterpart was 25.9 Å, whereas the distance without soluble counterpart was 20.7 Å (*p*-value = 0.0088, Mann-Whitney *U*) (Figure S5). This result may reflect the more facile exchange of domains that are not deeply entwined within the membrane protein structure.

**Figure 2:**
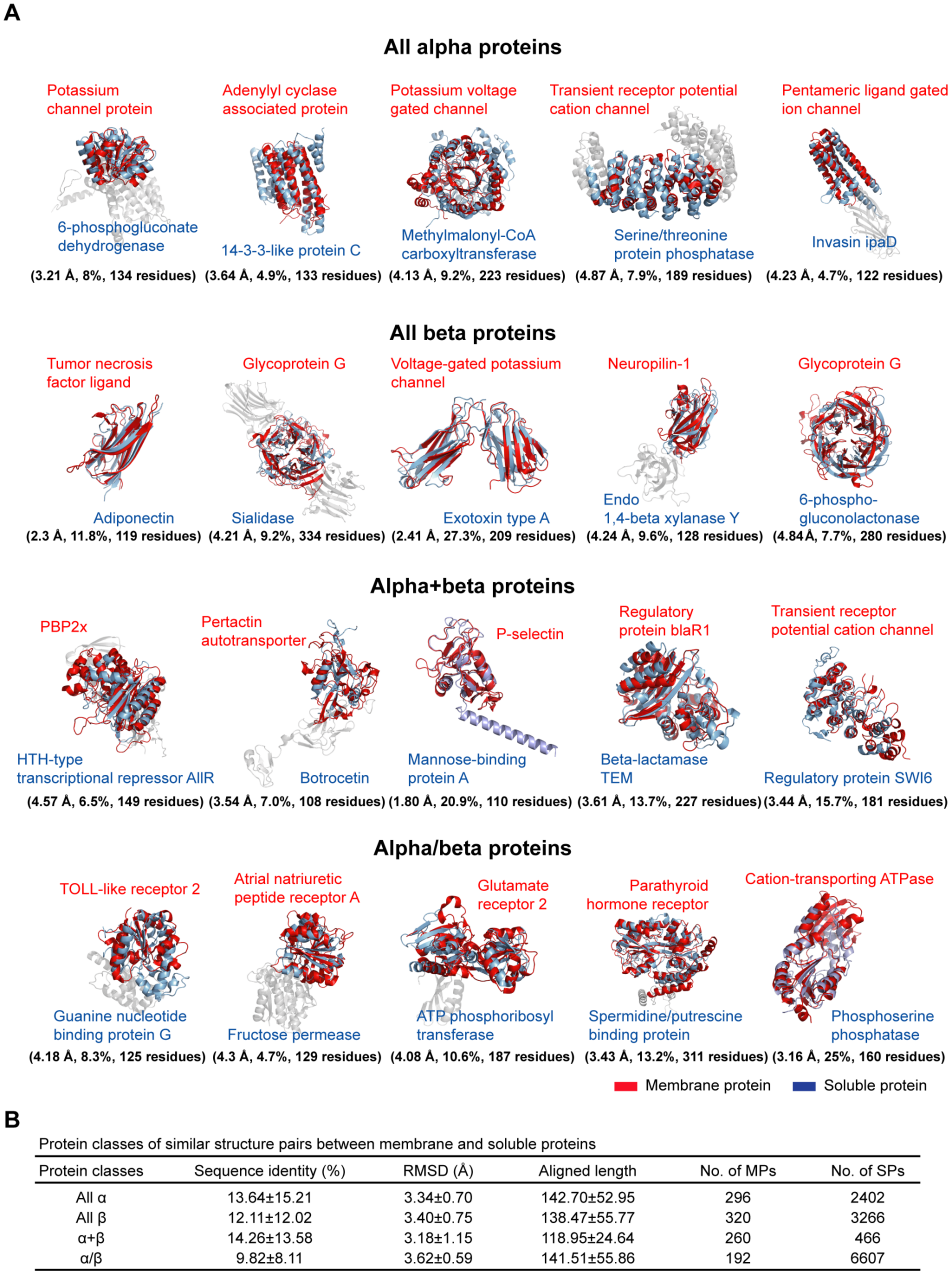
Structurally aligned pairs of membrane and soluble proteins. (A) All alpha, all beta, alpha+beta and alpha/beta classes from SCOP databases were represented. The RMSD, sequence identity, and aligned lengths of each pair are noted in parentheses. (B) Protein classes of similar structure pairs between membrane and soluble proteins.

The structural relatives do not appear to be restricted to any particular type of fold as they span many SCOP classes, including all alpha, all beta, alpha+beta and alpha/beta classes (Figure 2). The aligned pairs share 352 different fold types (Table S1) which is roughly a quarter of the 1,200 total fold types in SCOP [9]. These results indicate that diverse fold types performing various biological functions are shared between membrane and soluble proteins.

We conducted a comprehensive gene ontology analysis for 29% membrane proteins that have no counterpart in the soluble proteins. It turned out that these membrane proteins were GPCRs families and sensory receptors families (G-proteins coupled receptor protein signaling pathway; *p* = 6.78e-54, sensory perception of chemical stimulus; *p* = 3.15e-49, sensory perception of smell; *p* = 6.58e-48) (Table S2). They usually have short extra-membrane regions and tend not to share globular domains with soluble proteins [10].

### Domain exchange is particularly important for eukaryotes

**Figure 3:**
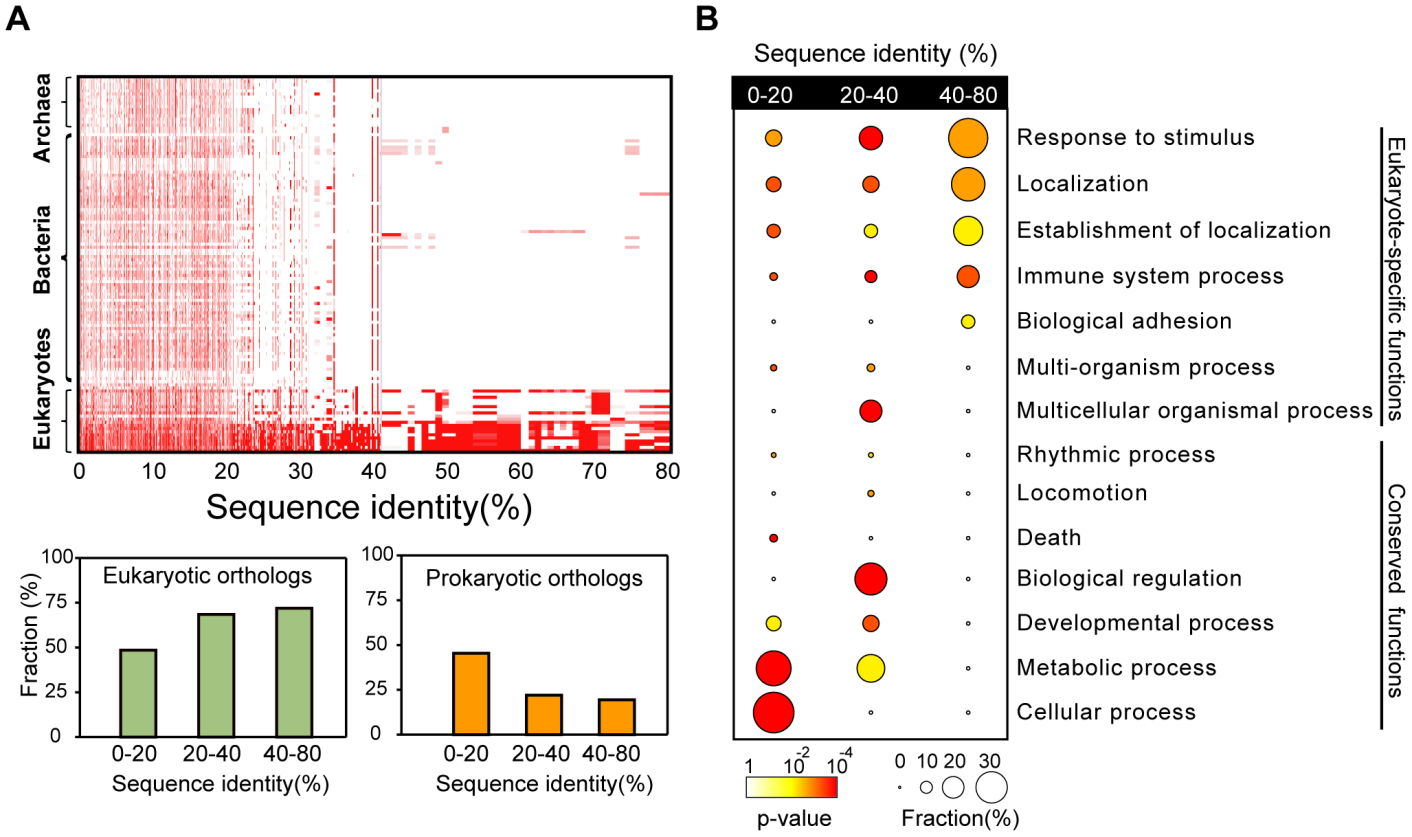
Phylogenetic and function enrichment analysis of the structure pairs of membrane and soluble proteins. (A) Phylogenetic distribution of soluble proteins that share similar structure with membrane proteins. Phylogenetic distribution was sorted by sequence identity of membrane and soluble proteins. For three groups divided by sequence identity between membrane and soluble proteins (low: 0–20%, medium: 20–40% and high: 40–80%), the fraction of eukaryotic and prokaryotic orthologues was represented. (B) Functional enrichment of membrane and soluble protein structure pairs. Three groups divided by their sequence identity were analyzed for enrichment of gene ontology. Circles of each functional term were colored by their *P*-value. The fraction of proteins which are included in each functional term is proportional to the diameter of the circles.

Figure 3A shows the distribution of sequence identities between soluble and membrane proteins grouped into archaea, bacterial and eukaryotes. High sequence identities reveal the soluble/membrane domain exchanges that occurred relatively recently in evolutionary history. The high sequence identities are dominated by eukaryotes, suggesting that many of the soluble/membrane protein exchanges in eukaryotes are relatively new developments. Figure 3B compares sequence identity distributions according to their functional ontologies. The basal cellular functions have the lowest sequence identities between membrane and soluble proteins, consistent with their ancient origin, whereas the more complex functions associated with eukaryotic organisms have higher sequence identities. These results suggest that as life became more complex, recombination of membrane and soluble proteins became more common and important.

### Can soluble protein annotations be used to illuminate membrane protein function?

**Figure 4:**
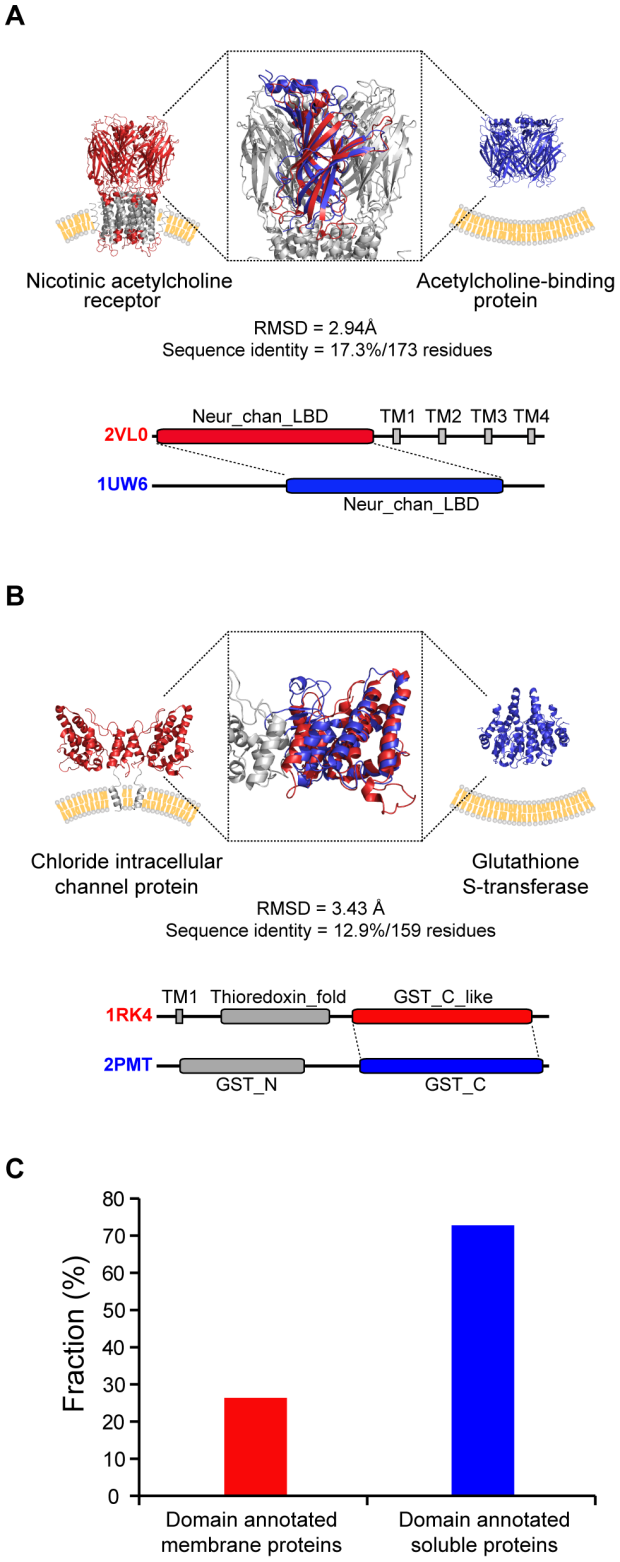
Shared domains between membrane and soluble proteins. (A) Nicotinic acetylcholine receptor and acetylcholine-binding protein. (B) Chloride intracellular channel protein and glutathione S-transferase. (C) Fraction of domain annotation found in membrane and soluble proteins.

Proteins that share similar domain structures often have similar functions even with very low sequence similarity. For example, the nicotinic acetylcholine receptor and acetylcholine-binding protein, which both bind acetylcholine, are found to share a domain that aligned with 2.94 Å RMSD over 173 residues, but shares only 17.3% sequence identity (Figure 4A) [11]. The chloride intracellular channel and glutathione S-transferase (GST) can be aligned with 3.43 Å RMSD over 159 residues, but share only 2.9% sequence identity (Figure 4B). Both proteins share a glutathione S-transferase function [12]–[14]. Thus, structural similarity can often suggest a functional similarity that cannot always be detected by sequence similarity. It therefore seems possible, given the extensive domain sharing noted above, to learn more about membrane protein functions by employing the annotations available for soluble proteins.

The soluble protein knowledge base could provide a rich source of information for membrane proteins as soluble proteins have generally been studied more extensively. Consistent with this history, only 26% of membrane protein domains that we found to align to soluble domains have annotated biochemical functions (109 of 414 proteins). In contrast, 72% (13,044 of 17,972 proteins) of soluble proteins that share domain structure with membrane proteins have domain annotation in the aligned regions (Figure 4C).

**Figure 5:**
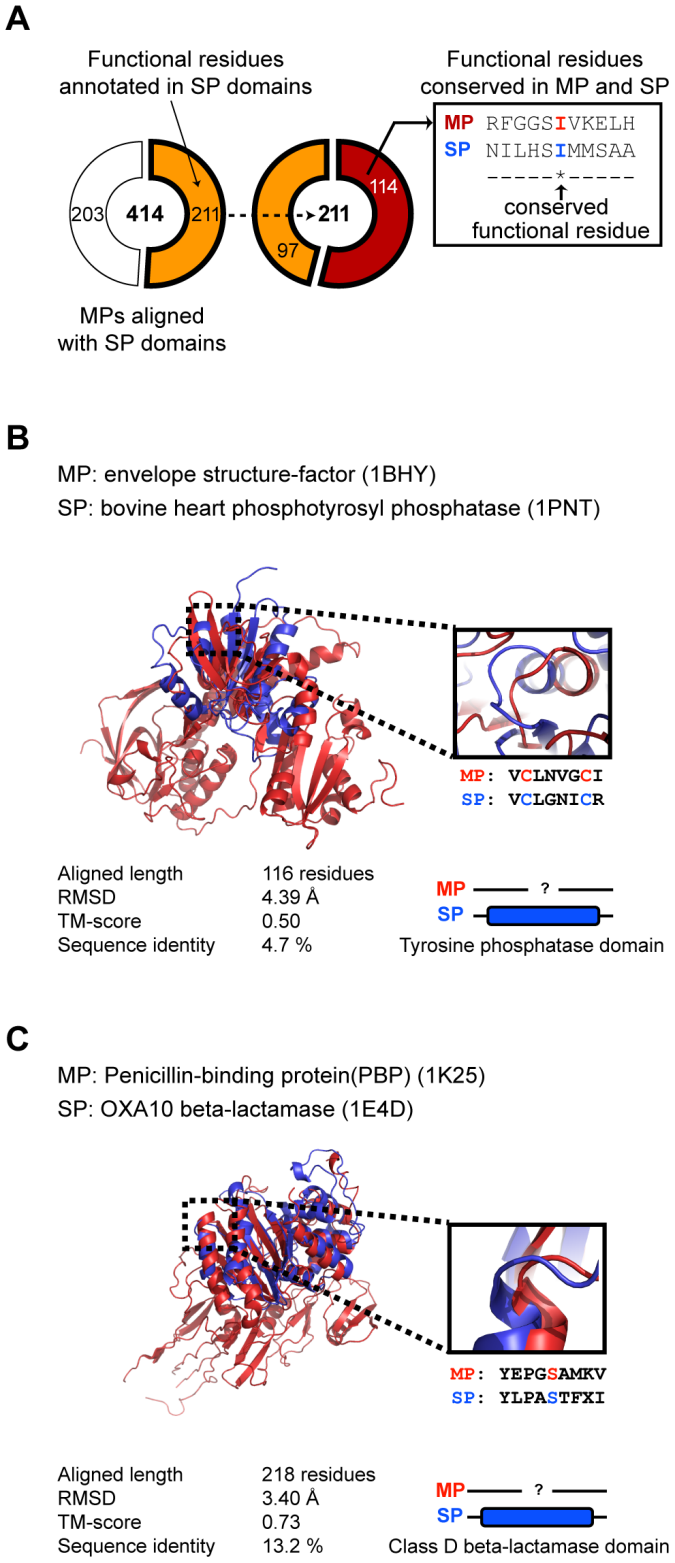
Functional residues conserved between membrane and soluble proteins. (A) Conserved catalytic sites between structurally aligned membrane and soluble protein domains. (B) Alignment of envelope structure-factor and phophotyrosyl phosphase. Catalytic sites are depicted on the structural alignment. (C) Alignment of penicillin-binding protein and OXA-10 beta-lactamase.

A common structure does not always imply a common function, however, so we examined the degree to which functional annotations might be transferrable from soluble proteins to membrane protein extracellular domains. To test the possibility of functional overlaps, we asked whether residues known to be critical for function were conserved in the structurally aligned pairs. For proteins with catalytic residues defined in the Catalytic Site Atlas (CSA) database [15] we found that 56% (114 of 211 proteins) of aligned structures share identical functional residues (Figure 5A). For example, the functional residues of bovine heart phosphotyrosyl phosphatase (soluble protein) are found to be conserved in envelope structure-factor (membrane protein), although their sequence identity is only 4.7% over 116 residues (Figure 5B). Bovine heart phosphotyrosyl phosphatase has a tyrosine phosphatase domain with the catalytic site residues, Cys12 and Cys17. Envelope structure-factor currently has no domain annotation, but the conserved catalytic sites as well as the aligned domain structures suggest that they may share a general biochemical function. Also, Penicillin-binding protein (membrane protein) and Oxa-10 β-lactamase (soluble protein) share identical functional residues although they only share 13.2% overall sequence identity over 218 residues (Figure 5C). Both apparently interact with β-lactam antibiotics. These results suggest that structure-guided alignments between membrane and soluble proteins can be useful for inferring unknown functions of extra-membrane domains.

We analyzed sequence identity of the first and second shell residues around the common functional sites compared to the rest of the residues. We defined the first shell residues as those within a distance of 5 Å of a known functional residue. The second shell residues were defined as the group of residues within 5 Å from the first shell residues. Sequence similarity scores were calculated using a PAM-250 matrix with the gap penalty of −11. We found that the first and second shell residues showed higher sequence similarity (Figure S6). Among the 471 structure pairs of membrane and soluble proteins, 412 structure pairs have higher sequence similarity at the first and second shell residues than other regions (Table S3). For example, the first and second shell residues around common functional sites of envelope structure-factor (1BHY) and bovine heart phosphotyrosyl phosphatase (1PNT) have higher sequence similarity than the rest of the residues (Figure S7A). The first and second shell residues of functional sites have a sequence similarity score of 123, whereas other residues have a sequence similarity score of 51.3. Also, the first and second shell residues of the functional sites of penicillin-binding protein (1K25) and Oxa-10 β-lactamase (1E4D) had higher sequence similarity than the rest of the residues (Figure S7).

We compared the functional annotations of membrane and soluble protein domains that share conserved functional residues. We discovered that 41% (28) of membrane protein domains share same the functional annotations with soluble domains and 31% (21) of membrane protein domains do not have functional annotation (Figure S8). Thus, these membrane protein functions can be inferred from the functional annotation of soluble proteins. But, 26% (18) of membrane protein domains turned out to have ambiguous functional annotations whose annotation were dissimilar but somewhat related. For example, membrane protein 1NRF has been annotated as beta-Lactamase/D-ala carboxypeptidase and soluble counterpart 2G2U has been annotated as beat-lactamase-inhibitor protein. We provide the list of functional annotation of membrane and soluble protein domains that share common functional residues (Table S4)

We examined how frequently shared domains between membrane and soluble proteins were found from same SCOP folds. Of 87 structurally similar domains, 60 (68.9%) extramembrane domains and soluble protein domain shared same SCOP folds, whereas 27 (31.1%) domains appeared in different SCOP folds (Figure S9A and Table S5). The number of fold types annotated for membrane proteins is much smaller than that of soluble proteins (Figure S9B). Specifically, structural pairs that share same SCOP fold were usually found from the extramembrane regions of membrane proteins. Meanwhile, structural pairs with different SCOP folds were mostly found from fold annotations assigned to whole membrane protein structures including both transmembrane and extramembrane regions.

We examined what kinds of membrane protein functions can be inferred from our work and to what extent. We classified membrane protein functions into 3 large families, such as receptors, transporters and enzymes, and divided into 16 sub families. We found that extramembrane domains shared between membrane and soluble proteins were mainly found from the enzyme family. Specifically, about 50% of the enzyme family of membrane proteins shares extramembrane domains with soluble counterparts, whereas less than 25% of the receptor family shares extramembrane domains with soluble counterparts (Figure S10). It suggests that function of membrane proteins in the enzyme family can be more likely inferred from the structural comparisons with soluble counterparts.

### Structure-guided sequence alignment of membrane and soluble proteins

The results described above indicate that membrane and soluble proteins extensively exchange domains and that soluble domain annotations can be useful for suggesting functions of the membrane domains. There are relatively few membrane protein structures, however, and the vast majority of structurally related proteins show little detectable sequence similarity. We therefore sought to expand the utility of the soluble domain structure database using both sequence and structural information.

**Figure 6:**
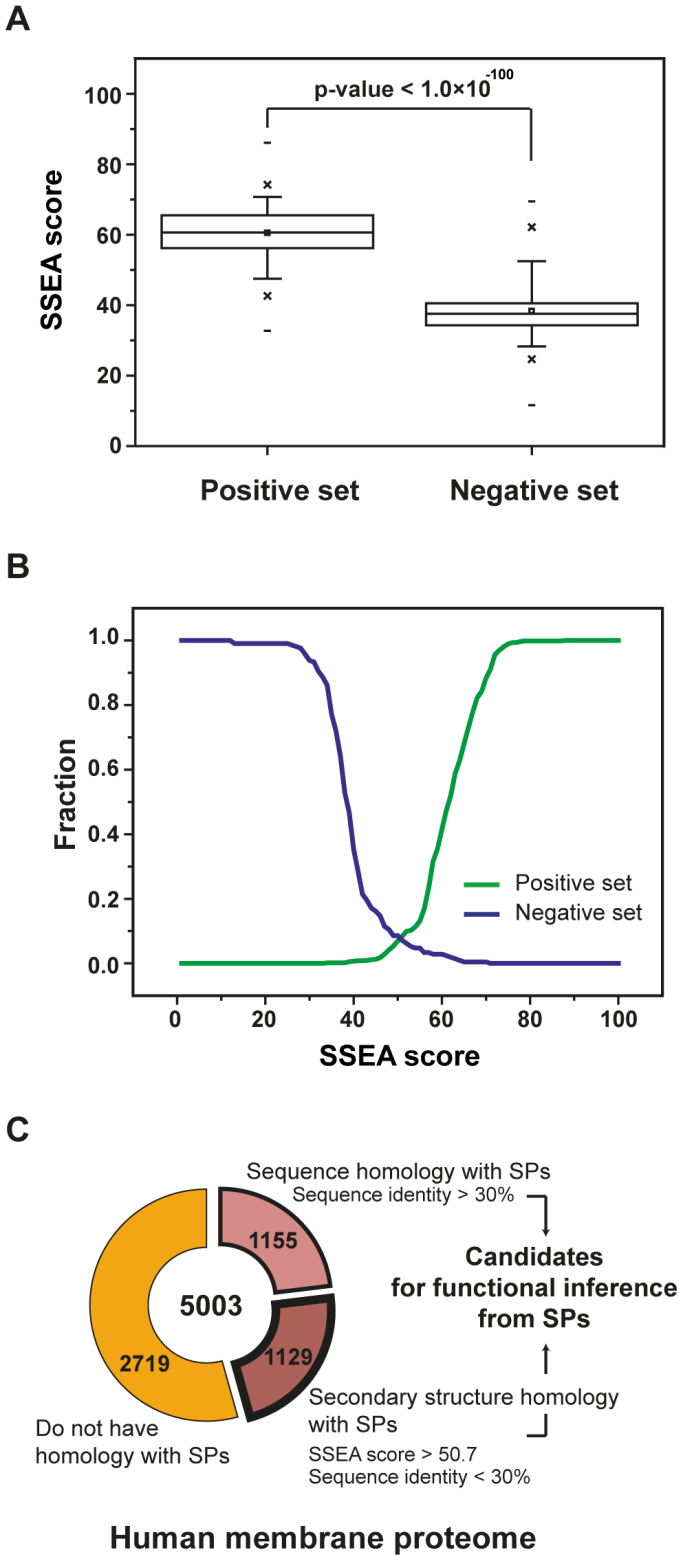
Training process of secondary structure element score to separate similar and dissimilar structure pairs between membrane and soluble proteins. (a) Distribution of the ssea scores of the positive set (similar structures) and the negative set (dissimilar structures) of membrane and soluble proteins. (b) SSEA score to filter the positives and the negatives was set to 50. (c) Fraction of membrane protein sequences that have structural homology with soluble protein structures.

To detect distant relationships that are not apparent by sequence similarity alone, we employed the secondary structure element alignment method (SSEA) [16]. To test the effectiveness of the SSEA method for detecting distant relationships and to identify appropriate cutoffs, we generated training sets. A positive set included 923 similar membrane and soluble protein structures with less than 5 Å RMSD and sequence identity ranging from 5 to 15%. The negative set included 210 dissimilar structure pairs with greater than 10 Å RMSD and sequence identity ranging from 5 to 15%. As shown in Figure 6A and 6B, the SSEA method can effectively separate the two training sets at an SSEA score of 50 (*P*-value<1.0×10^−100^) [16]. Moreover, we calculated the probability of finding structure pairs with RMSD<5 Å and discovered that it was dramatically increased over SSEA score 50 (Figure S11). Thus, the SSEA method can allow us to detect many more relationships than would be possible by sequence similarity alone.

### Application to the human proteome

We searched for soluble/membrane protein structural relationships in the human proteome (Figure S12). Of 5003 membrane proteins in the human genome, we found that 1,155 showed clear sequence similarity to soluble proteins of known structure. Moreover, of 1,155 TM proteins, 449 TM proteins were aligned with soluble domains bearing SwissProt domain annotations (Table S6). Employing the SSEA method, we could assign an additional 1,129 proteins as probable relatives of soluble proteins of known structure. Thus, a detectable structural relative exists for ∼45% of the membrane proteins in the human genome (Figure 6C).

An example of the type of information that can be derived is shown in Figure S13. Monoacylglycerol lipase ABHD6 (membrane protein) and epoxide hydrolase 2 (soluble protein) aligned well with the SSEA score of 66.91 and shared experimentally verified active site residues, Asp495 and His523, suggesting that these proteins may have a common hydrolase function. We believe the list of identified structural relationships will be a useful resource for developing functional hypotheses and the list is provided at sbi.postech.ac.kr/emdmp.

## Discussion

Our results show that membrane proteins quite commonly acquire or share functions by domain exchange with soluble proteins. There has been a controversy over whether membrane or soluble proteins have emerged first during evolution and several reports support the idea that membrane proteins may have come first [17]–[19]. They argue that membrane proteins require less extensive sequence optimization for folding than soluble proteins because they reside in a more restrictive membrane environment. However, we suggest that the evolutionary paths of membrane proteins might be more diverse. For example, we found that a soluble protein, 3-hydroxy-3-methylglutaryl-CoA (HMG-CoA) reductase, exists in all three kingdoms, whereas the membrane form of HMG-CoA reductase only exists in eukaryotic species (Figure S14A) [20], [21]. This suggests that the evolutionary origin of HMG-CoA reductase may be a soluble form and the membrane form was created by acquiring transmembrane domains. Alternatively, the membrane variants in prokaryotes could have been lost at some point in evolution. On the other hand, acetylcholine-binding proteins may have emerged from eukaryotic species by losing the transmembrane domains of nicotinic acetylcholine receptors (Figure 4A). Nicotinic acetylcholine receptors exist in all three kingdoms, but acetylcholine-binding proteins only exist in eukaryotes. Thus, it seems reasonable to suggest that membrane and soluble proteins exchange domains and functionalities in both directions over the course of evolution (Figure S14B). The fact that the more recent exchanges have occurred in eukaryotes suggests that this became a particularly important evolutionary mechanism as life became more complex.

Regardless of the evolutionary origins, it is clear that many membrane and soluble proteins share structural similarity. Similar folds do not always imply similar function, but in many cases, structural similarities of proteins have been used to discover functional similarities [22]–[25]. This is based on the notion that sequence and structure similarities between gene products infer functional similarities [26]–[28]. We can therefore utilize structural and functional information obtained from one class to report on the other.

## Materials and Methods

### Data sets of membrane and soluble protein structures

We collected 558 membrane and 43547 soluble protein structures from the PDB library [29]. We included only structures solved by X-ray and NMR, and excluded structures solved by EM (electron microscopy and cryo-electron diffraction), Fiber (fiber diffraction), IR (infrared spectroscopy), Model (predicted models), Neutron (neutron diffraction). Only experimentally confirmed membrane protein structures from the SwissProt and PDB databases were included. Proteins annotated as single-/multi-pass membrane proteins or membrane proteins were included, but peripheral membrane proteins were excluded. We collected soluble protein structures by excluding membrane proteins and putative membrane proteins. The SCOP database (release 1.75) was used to examine the fold and class diversity of structures. The current SCOP database lists only 58 folds of membrane proteins, whereas more than 1000 folds are listed for soluble proteins.

### Pair-wise structure comparisons between membrane and soluble proteins

We compared structure pairs of membrane and soluble proteins using TM-align, a structure comparison algorithm which uses dynamic programming and alignment confidence score rotation matrix [6]. TM-align is a suitable tool for large-scale structural comparisons. The calculation time of TM-align was faster than other structure alignment programs, such as CE and DALI [30], [31]. The average CPU time per pair by TM-align was 0.3s, which was 40 time faster than CE (*P*-value = 1.65e-56, *t*-test). For the calculation, we randomly selected structure pairs of membrane and soluble proteins 1,000 times. Calculations were performed on 2.66 GHz hexa core CPU LINUX machine. We compared structural superimposition of TM-align with other tools by using 10,000 random pairs between membrane and soluble proteins. We found that CE and DALI gave equivalent results of structural alignments compared with TM-align. Particularly, RMSD values from each tool are highly correlated for the same structure pairs (Figure S1A and S1B).

We applied a strict cutoff of RMSD, aligned length, and alignment confidence score to select only significantly aligned structure pairs between membrane and soluble proteins. Structure pairs with RMSD<5 Å, aligned length >100 residues, and alignment confidence score (TM-score) >0.5 were selected. Structural alignments of relatively shorter sequence (less than 100 residues) gave somewhat dissimilar results (Figure S1C and S1D) when we applied different tools. Thus, we chose aligned length >100 residues as a length threshold. These selection criteria have been found to filter out dissimilar structures in other high-throughput structural comparison studies [1], [6], [32], [33]. We applied PDBTM database to measure whether structural similarity occurred in the extramembrane or transmembrane regions of membrane proteins. Membrane proteins that shared structural similarity within transmembrane region were removed. Furthermore, structure pairs that have several disconnected extramembrane loops were removed since these short loops cannot act as independent domains. We mapped the topology information (i.e. inside and outside regions) of membrane proteins onto the structural alignment results using TMHMM [34], [35]. The procedure of structure comparisons between membrane and soluble proteins is described in Figure S15.

### Class, fold and domain identification of aligned structural pairs

We classified structurally similar membrane and soluble proteins into four classes; all alpha, all beta, alpha+beta, and alpha/beta based on SCOP classifications [9]. SCOP database is a comprehensive ordering of all proteins of known structures according to their structural relationships. Because structural information of membrane proteins is lacking, we utilized class information of soluble proteins to identify the class of structurally aligned membrane and soluble protein pairs. We used the domain information from the SCOP database to assign domain boundaries of the structurally aligned regions of membrane and soluble proteins. We assigned a domain annotation if an aligned region covered more than the 90% of domain length.

### Analysis of phylogenetic profile and functional enrichment

We used 120 fully sequenced genomes of archaea, bacteria and eukaryotes to compare orthologs of soluble proteins aligned with membrane proteins. The 120 genomes are comprised of 9 archaea, 80 bacteria and 21 eukaryotic species. InParanoid was used to detect the orthologs of query proteins [36]. For functional enrichment analysis, we used a function annotation tool, DAVID [37]. Among the 31 biological process terms in the level 1 of gene ontology hierarchy, we found 14 terms in which at least one protein is involved.

### Measurement of the membrane distance of extramembrane domains

We collected 504 extramembrane domains which have soluble counterparts and 102 extramembrane domains which don't have soluble counterparts. We transformed molecular coordinate of each membrane protein structures to be parallel with the membrane plane by using the PDBTM database. Membrane distance of extramembrane domains was measured between the average of all the coordinates of domains and the surface of membrane bilayer.

### Structure-guided sequence alignment using the secondary structure element score

We applied the SSEA method that can detect possible structural homology in the absence of strong sequence similarity by including secondary structure pattern information [16]. Secondary structures of membrane and soluble proteins were predicted by PSIPRED [38]. To set a reliable cut-off value for the structural comparisons, we evaluated SSEA score based on a positive and a negative set. The positive set includes structure pairs of membrane and soluble proteins with <5 Å RMSD and sequence identity range from 5 to 15%. The negative set includes dissimilar structure pairs of membrane and soluble proteins with >10 Å RMSD and sequence identity range from 5 to 15%. We selected 100 pairs from each positive and negative set by random sampling. We compared the SSEA score of these pairs from each group and repeated the process 1,000 times. We found that SSEA score of 50 works best for separating the positive set from the negative set (*P*-value<1.0×10^−100^; Figure 6AB). Furthermore, we analyzed the correlation between SSEA score and the probability of finding structure pairs with RMSD <5 Å (Figure S11). To calculate the probability, we randomly selected 10,000 structure pairs of membrane and soluble proteins from all ranges of RMSD values. We found that the probability of finding structure pairs with RMSD <5 Å was dramatically increased with an SSEA score over 50.

We compared the structure-guided sequence alignment results of SSEA with HHpred [39]. We found that SSEA and HHpred gave similar alignment results except for the positive prediction rates. SSEA provided more positive sets than HHpred for the structural comparisons (Figure S16). The domain structures shared between membrane and soluble proteins usually have low sequence identity and it has been shown that the HMM method tends to have difficulties detecting distant homologs [40], [41]. Therefore, for the comparisons of membrane and soluble domains with very low sequence identity, the SSEA method was chosen.

## Supporting Information

Figure S1 **Comparison of structure superimposition by TM-align and other tools.** (A) Structural comparisons between TM-align and CE. (B) Structural comparisons between TM-align and DALI. Orange dots and the percentage represent the similar structure pairs with RMSD <5 Å from both tools. Gray dots represent the structure pairs with RMSD <5 Å by TM-align only. (A),(B) Structure comparison result for the structure pairs with RMSD <5 Å and aligned length >100 residues. (C) Structural comparisons between TM-align and CE. (D) Structural comparisons between TM-align and DALI. (C),(D) Structure comparison result for the structure pairs with RMSD <5 Å and aligned length <100 residues.
(TIF)

Figure S2 **Structurally aligned membrane proteins after removing redundant sequences at a threshold of 30% sequence identity.**
(TIF)

Figure S3 **Web-server for the structure alignment of membrane and soluble proteins.** (A) Main data page. Users can input PDB ID or Pfam domain names and download structure alignment data. (B) The main output page of web-server. Structure comparison data, such as PDB IDs, chain IDs of membrane and soluble proteins, aligned region, RMSD, TM-score, sequence identity, i-m-o topology and Pfam domains of aligned regions are provided. (C) Alignment results of membrane and soluble proteins.
(TIF)

Figure S4 **Aligned lengths of the extramembrane domains located at the outside and inside regions of membrane proteins.**
(TIF)

Figure S5 **Membrane distances of extramembrane domains with or without soluble counterparts.**
(TIF)

Figure S6 **Difference of sequence similarity scores between the first/second shell residues and the rest of the functional residues.** Sequence similarity scores were calculated from 471 structural pairs.
(TIF)

Figure S7 **Sequence similarity scores of the first and second shell residues around the functional sites.** (A) Envelope structure-factor (1BHY) and bovine heart phosphotyrosyl phosphatase (1PNT). (B) penicillin-binding protein (1K25) and Oxa-10 β-lactamase (1E4D)
(TIF)

Figure S8 **Functional annotations of the structurally aligned membrane and soluble protein that share conserved functional residues.**
(TIF)

Figure S9 **Shared SCOP folds of membrane and soluble proteins.** (A) Fraction of shared domains in the same and different SCOP folds of structurally aligned membrane and soluble proteins. (B) SCOP fold annotations of membrane and soluble proteins.
(TIF)

Figure S10 **Fraction of membrane protein families that share extramembrane domains with soluble counterparts.** (A) Three membrane protein families that share extramembrane domains with soluble counterpart. (B) Sixteen membrane protein sub-families that share extramembrane domains with soluble counterpart.
(TIF)

Figure S11 **Probability of finding structure pairs with RMSD <5Å and aligned length >100 residues by SSEA scores.**
(TIF)

Figure S12 **Procedure for structure-guided sequence alignment.** Secondary structure element alignment was applied to select structurally comparable sequences of membrane and soluble proteins.
(TIF)

Figure S13 **Alignment of secondary structure elements and functional residues between monoacylglycerol lipase ABHD6 and epoxide hydrolase 2.** (A) Secondary structure comparison between monoacylglycerol lipase ABHD6 (membrane protein) and epoxide hydrolase 2 (soluble protein). (B) Conserved functional residues of epoxide hydrolase 2 and monoacylglycerol lipase ABHD6 were highlighted (yellow box).
(TIF)

Figure S14 **Phylogenetic profiles of membrane and soluble proteins that share extramembrane domains.** (A) Phylogenetic profiles of the membrane and soluble forms of 3-hydroxy-3-methylglutaryl-CoA HMG-CoA reductase. (B) Phylogenetic profiles of nicotinic acetylcholine receptor and acetylcholine-binding protein.
(TIF)

Figure S15 **Procedure for the structure alignment of membrane and soluble proteins.**
(TIF)

Figure S16 **Comparison of structure-guided sequence alignment results by SSEA and HHpred.** (A) Prediction results of SSEA score. (B) Prediction results of HHpred.
(TIF)

Table S1 **Fold types of similar structure pairs between membrane and soluble proteins.**
(DOC)

Table S2 **GO enrichment of membrane proteins that have not exchanged domains with soluble proteins.**
(DOC)

Table S3 **Sequence similarity scores of the first and second shell residues around common functional sites.**
(XLS)

Table S4 **Functional annotation of membrane and soluble protein domains that share conserved functional residues.**
(XLS)

Table S5 **Common SCOP folds shared by membrane and soluble proteins.**
(DOC)

Table S6 **SwissProt domains of membrane proteins that share sequence similarity with soluble proteins.**
(DOC)
