# Supplementary figures and images for: Rampant Exchange of the Structure and Function of Extramembrane Domains between Membrane and Water Soluble Proteins
Source: PLoS Comput Biol. 2013 Mar 21;9(3):e1002997. doi: 10.1371/journal.pcbi.1002997 (PMC3605051; doi:10.1371/journal.pcbi.1002997)

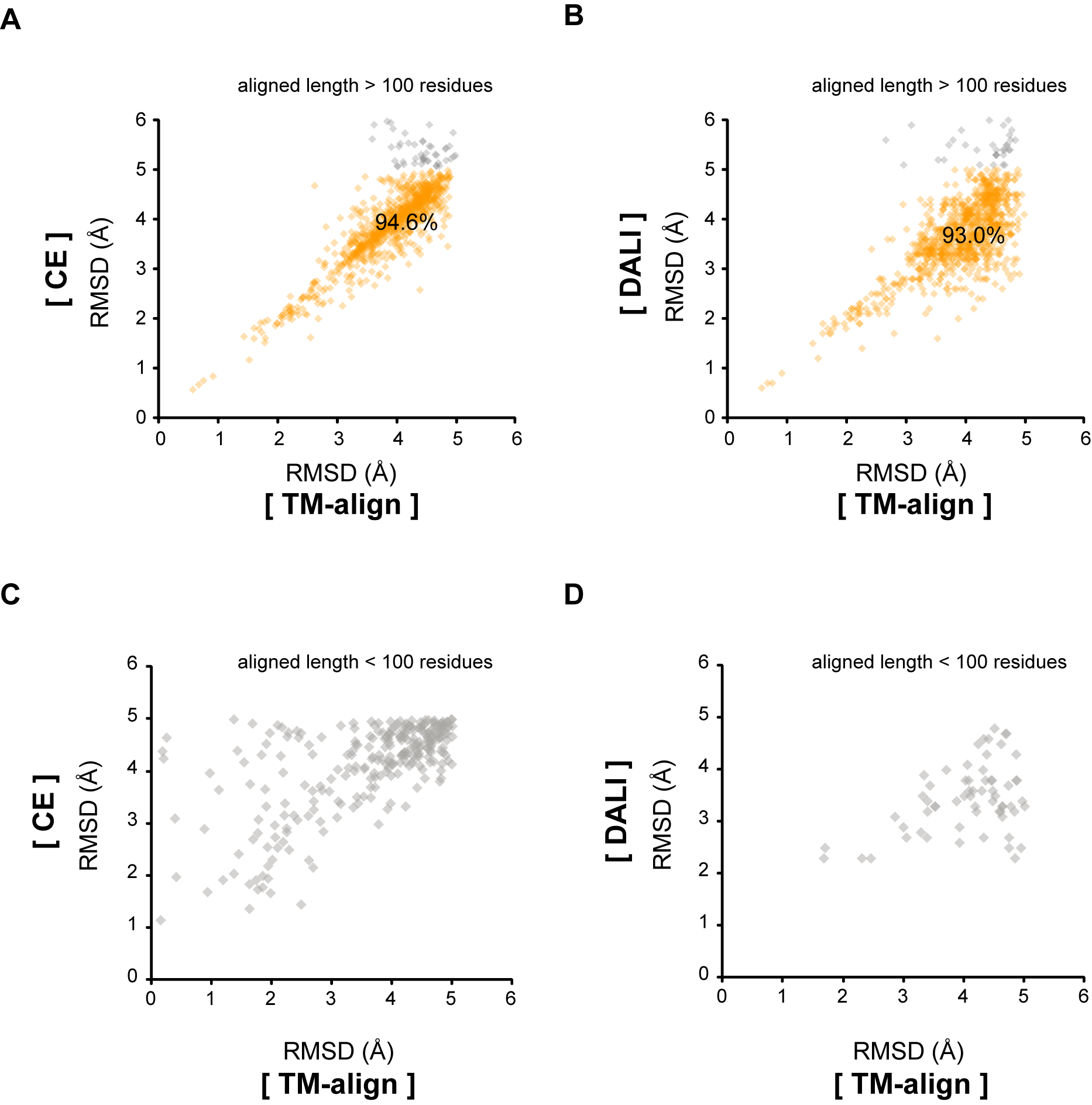

Supplement: Figure S1 — Comparison of structure superimposition by TM-align and other tools. (A) Structural comparisons between TM-align and CE. (B) Structural comparisons between TM-align and DALI. Orange dots and the percentage represent the similar structure pairs with RMSD <5 Å from both tools. Gray dots represent the structure pairs with RMSD <5 Å by TM-align only. (A),(B) Structure comparison result for the structure pairs with RMSD <5 Å and aligned length >100 residues. (C) Structural comparisons between TM-align and CE. (D) Structural comparisons between TM-align and DALI. (C),(D) Structure comparison result for the structure pairs with RMSD <5 Å and aligned length <100 residues. (TIF) [file pcbi.1002997.s001.tif]

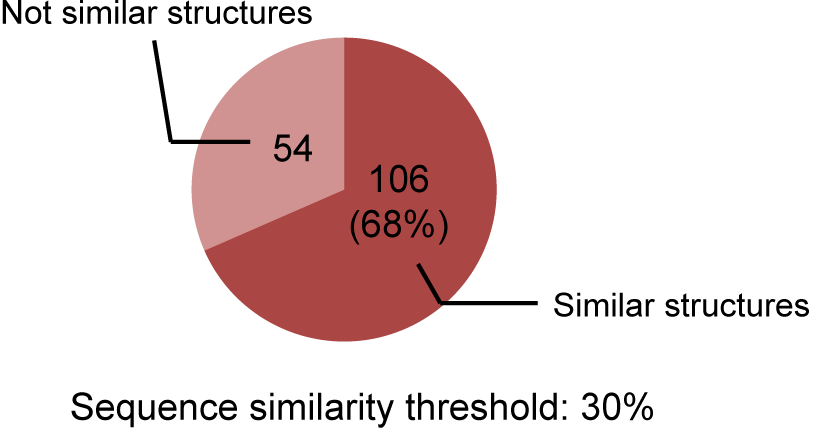

Supplement: Figure S2 — Structurally aligned membrane proteins after removing redundant sequences at a threshold of 30% sequence identity. (TIF) [file pcbi.1002997.s002.tif]

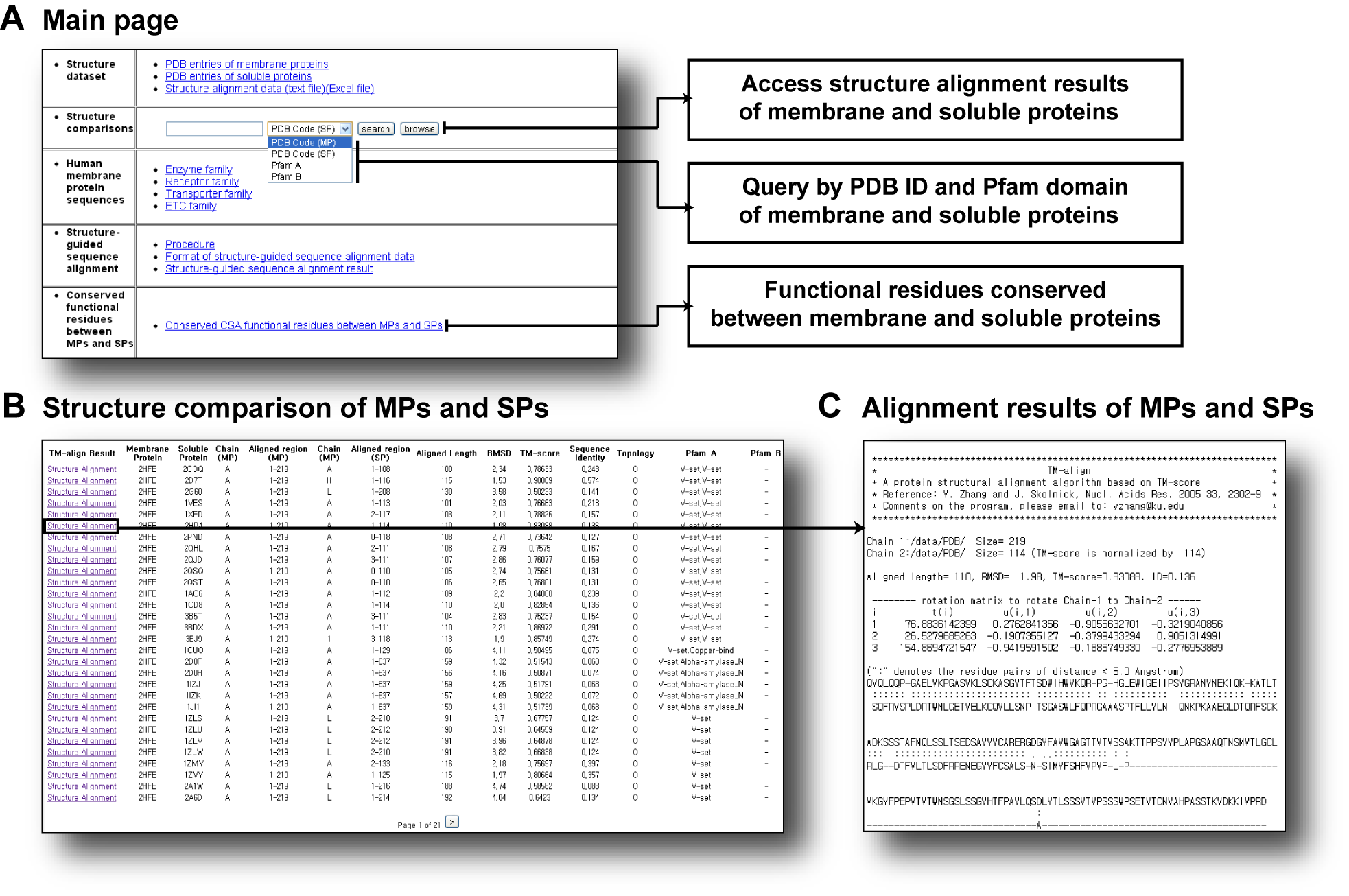

Supplement: Figure S3 — Web-server for the structure alignment of membrane and soluble proteins. (A) Main data page. Users can input PDB ID or Pfam domain names and download structure alignment data. (B) The main output page of web-server. Structure comparison data, such as PDB IDs, chain IDs of membrane and soluble proteins, aligned region, RMSD, TM-score, sequence identity, i-m-o topology and Pfam domains of aligned regions are provided. (C) Alignment results of membrane and soluble proteins. (TIF) [file pcbi.1002997.s003.tif]

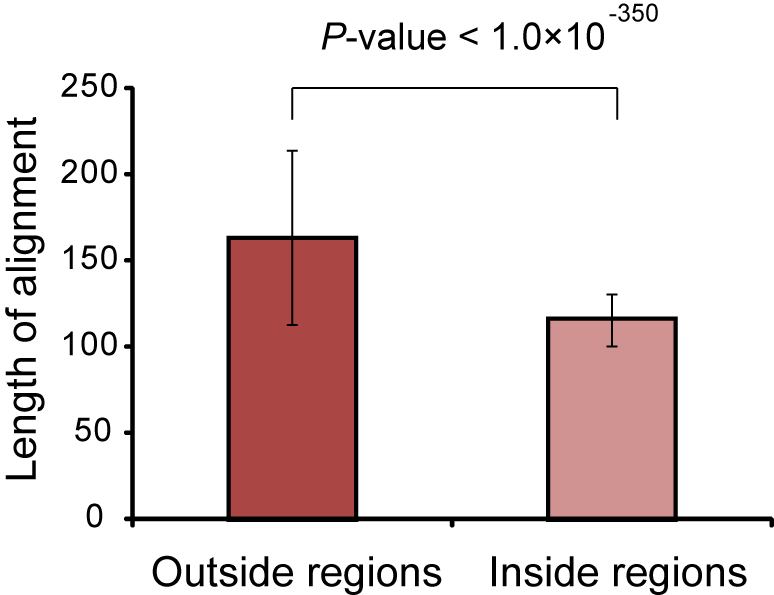

Supplement: Figure S4 — Aligned lengths of the extramembrane domains located at the outside and inside regions of membrane proteins. (TIF) [file pcbi.1002997.s004.tif]

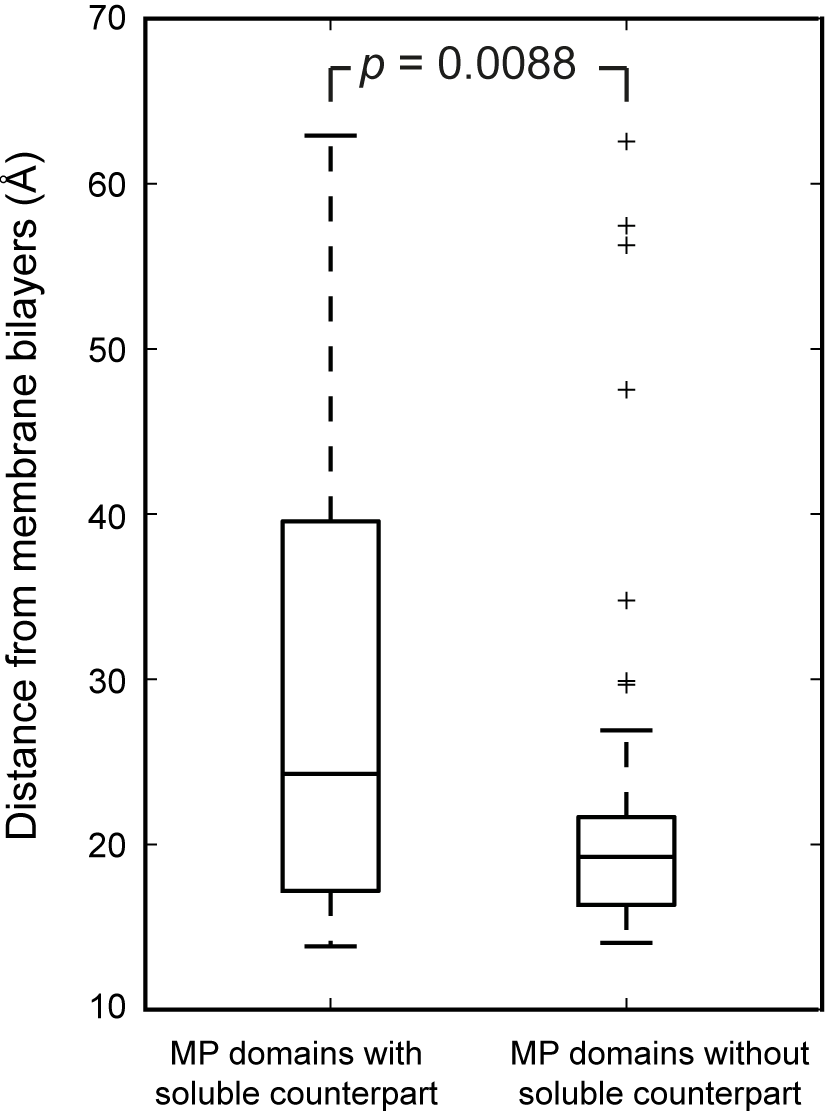

Supplement: Figure S5 — Membrane distances of extramembrane domains with or without soluble counterparts. (TIF) [file pcbi.1002997.s005.tif]

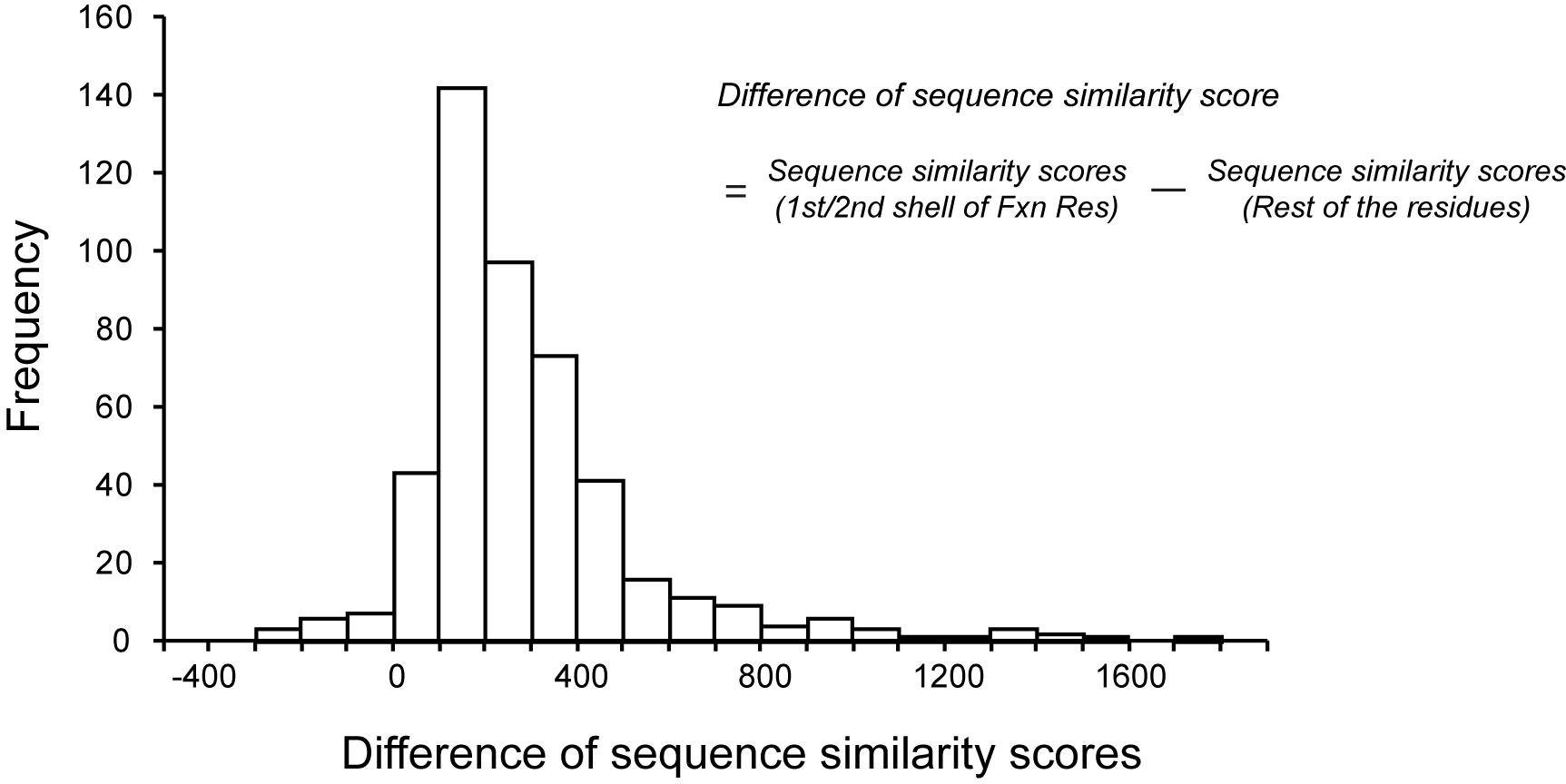

Supplement: Figure S6 — Difference of sequence similarity scores between the first/second shell residues and the rest of the functional residues. Sequence similarity scores were calculated from 471 structural pairs. (TIF) [file pcbi.1002997.s006.tif]

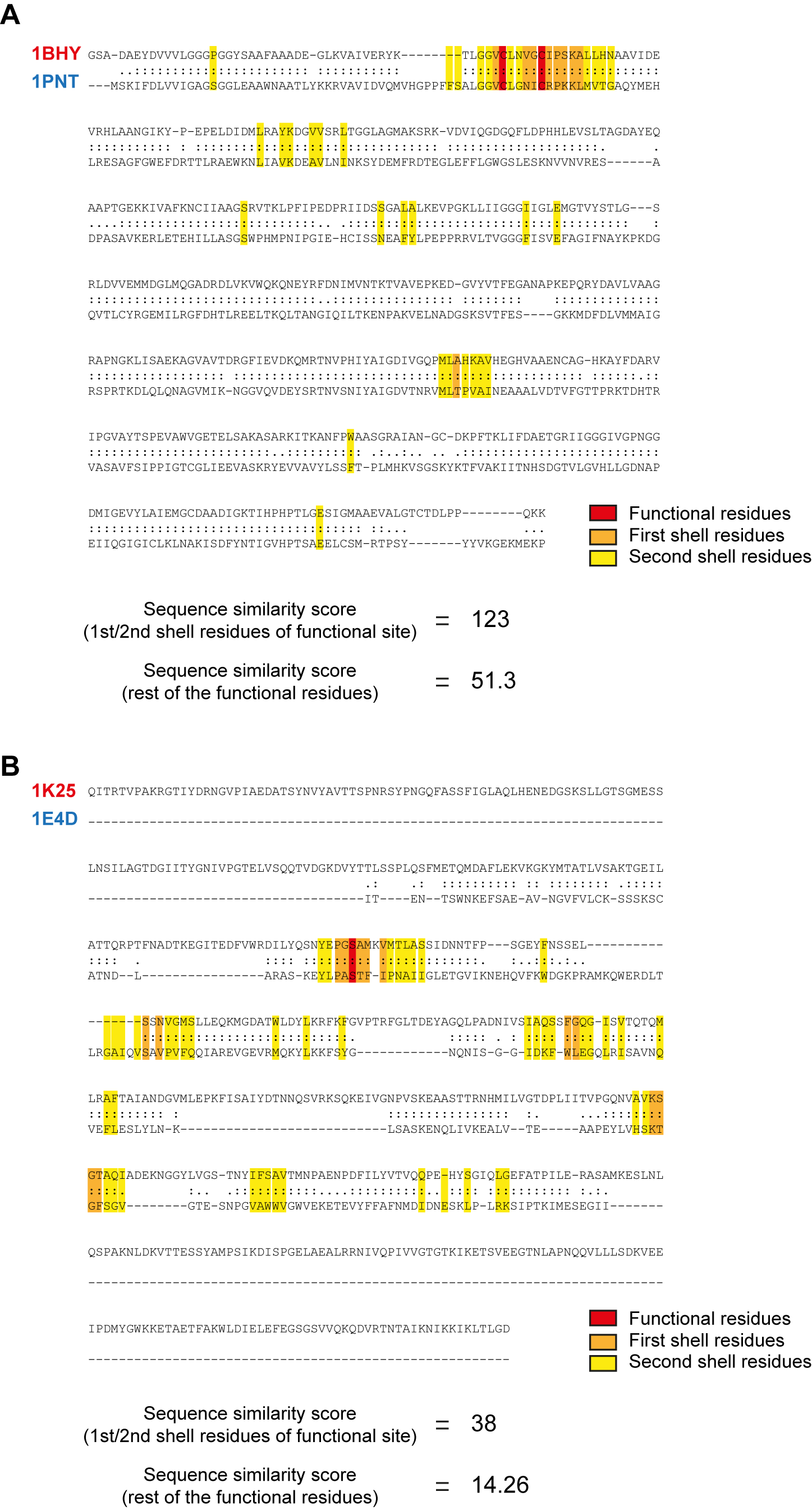

Supplement: Figure S7 — Sequence similarity scores of the first and second shell residues around the functional sites. (A) Envelope structure-factor (1BHY) and bovine heart phosphotyrosyl phosphatase (1PNT). (B) penicillin-binding protein (1K25) and Oxa-10 β-lactamase (1E4D) (TIF) [file pcbi.1002997.s007.tif]

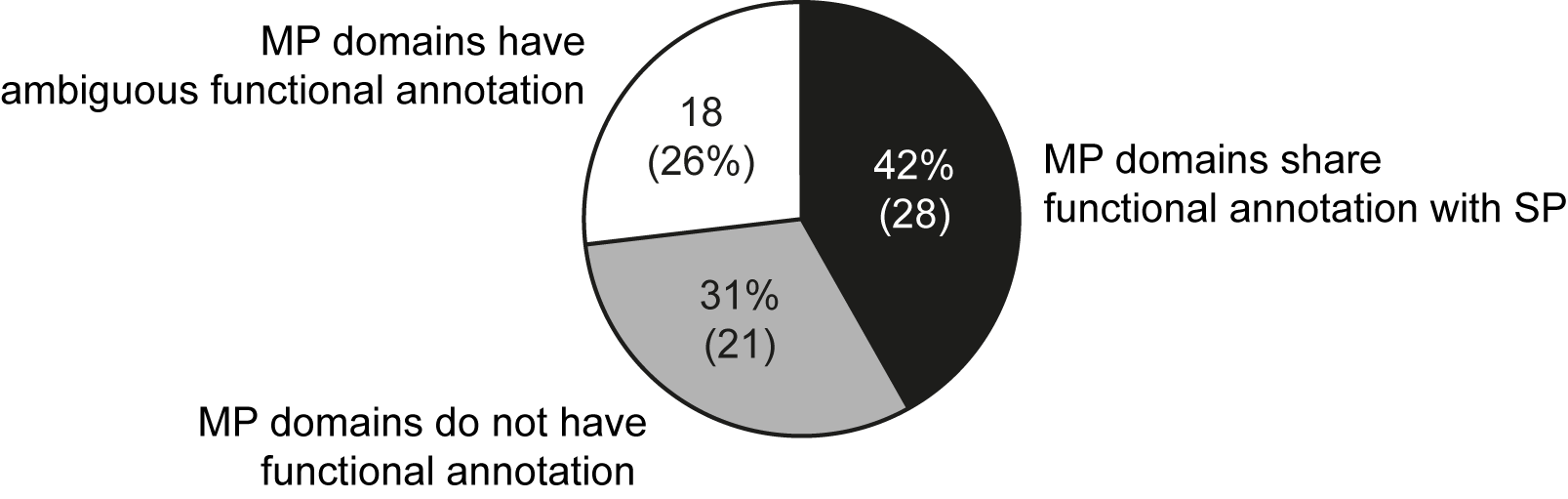

Supplement: Figure S8 — Functional annotations of the structurally aligned membrane and soluble protein that share conserved functional residues. (TIF) [file pcbi.1002997.s008.tif]

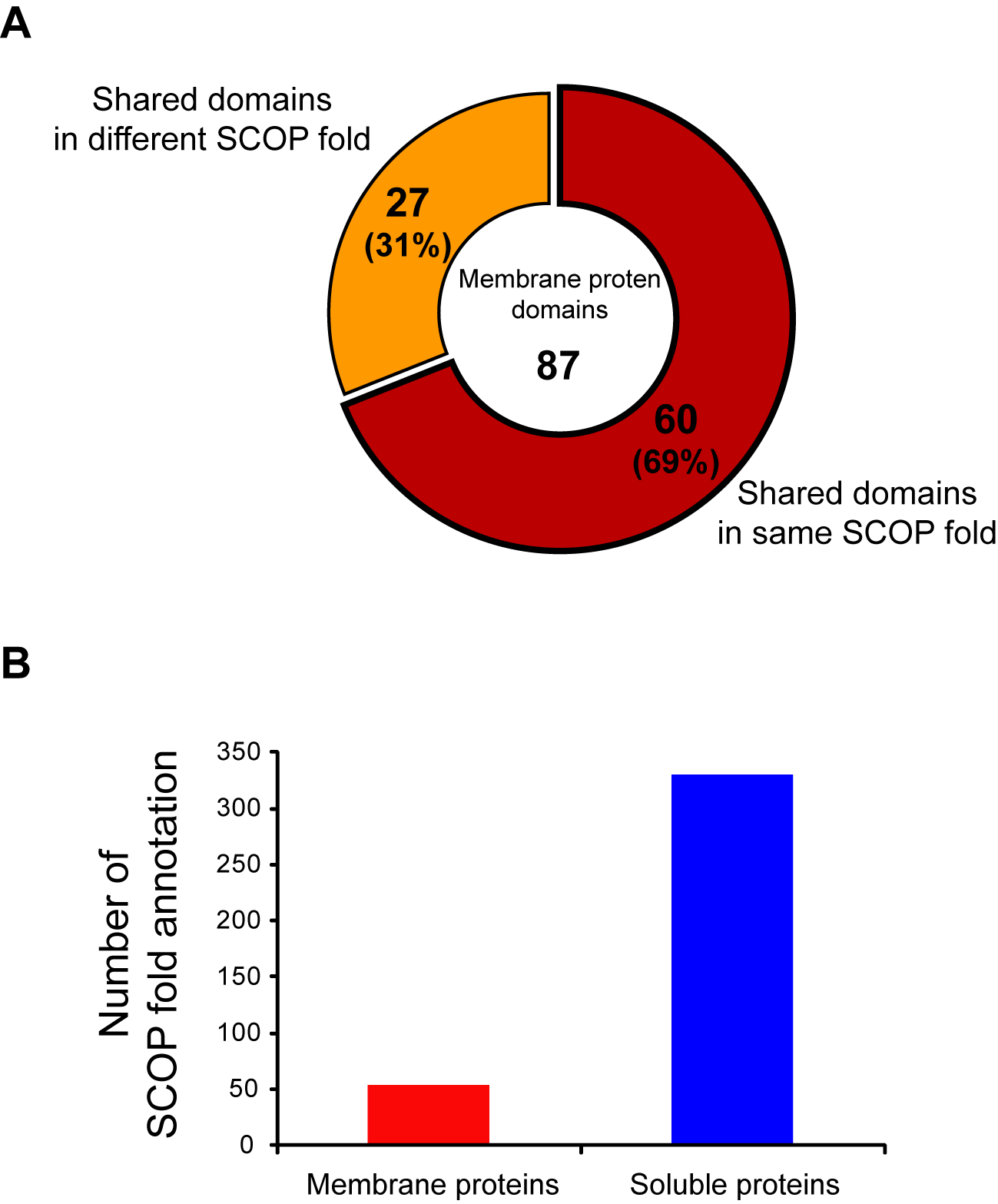

Supplement: Figure S9 — Shared SCOP folds of membrane and soluble proteins. (A) Fraction of shared domains in the same and different SCOP folds of structurally aligned membrane and soluble proteins. (B) SCOP fold annotations of membrane and soluble proteins. (TIF) [file pcbi.1002997.s009.tif]

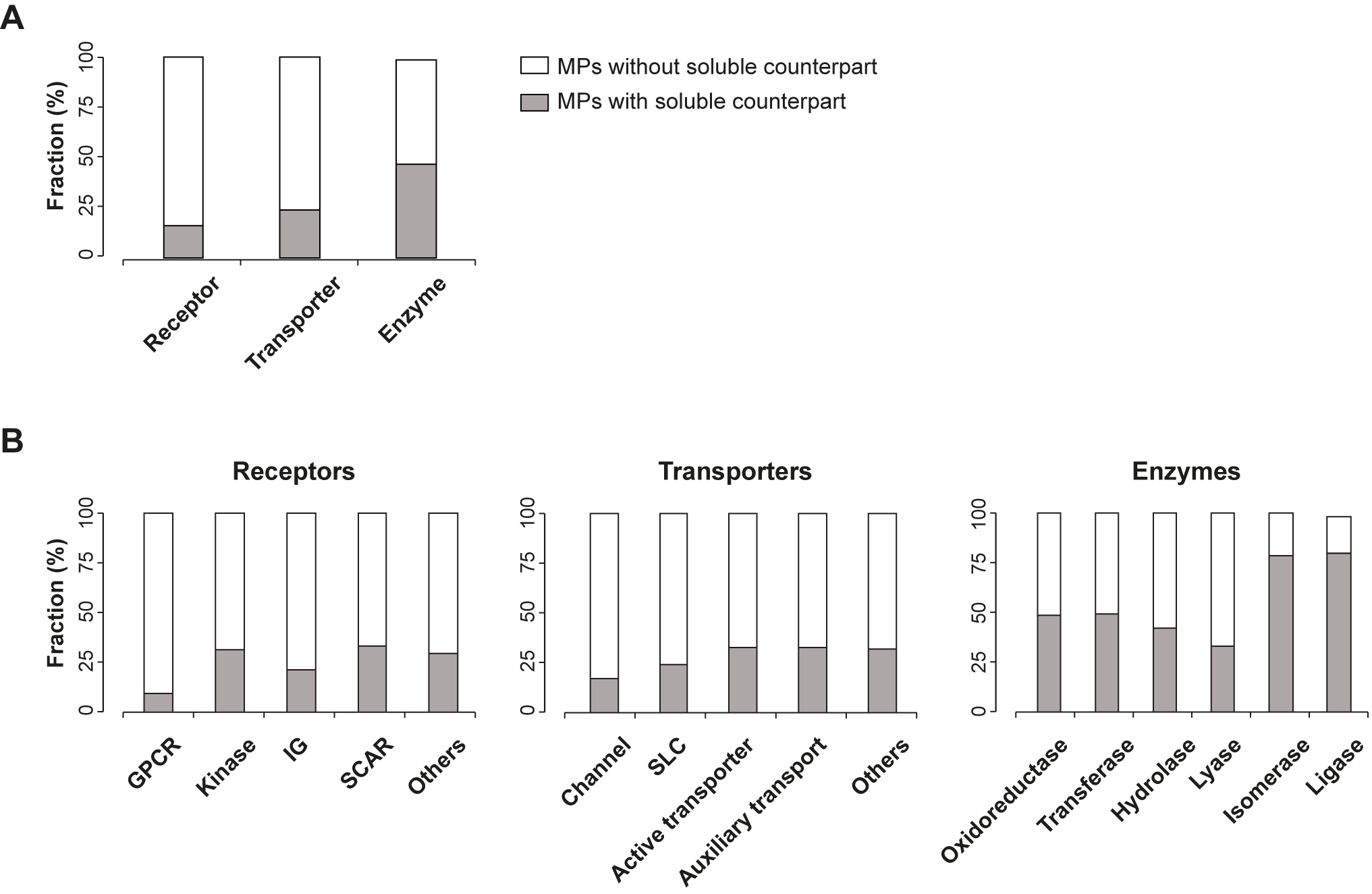

Supplement: Figure S10 — Fraction of membrane protein families that share extramembrane domains with soluble counterparts. (A) Three membrane protein families that share extramembrane domains with soluble counterpart. (B) Sixteen membrane protein sub-families that share extramembrane domains with soluble counterpart. (TIF) [file pcbi.1002997.s010.tif]

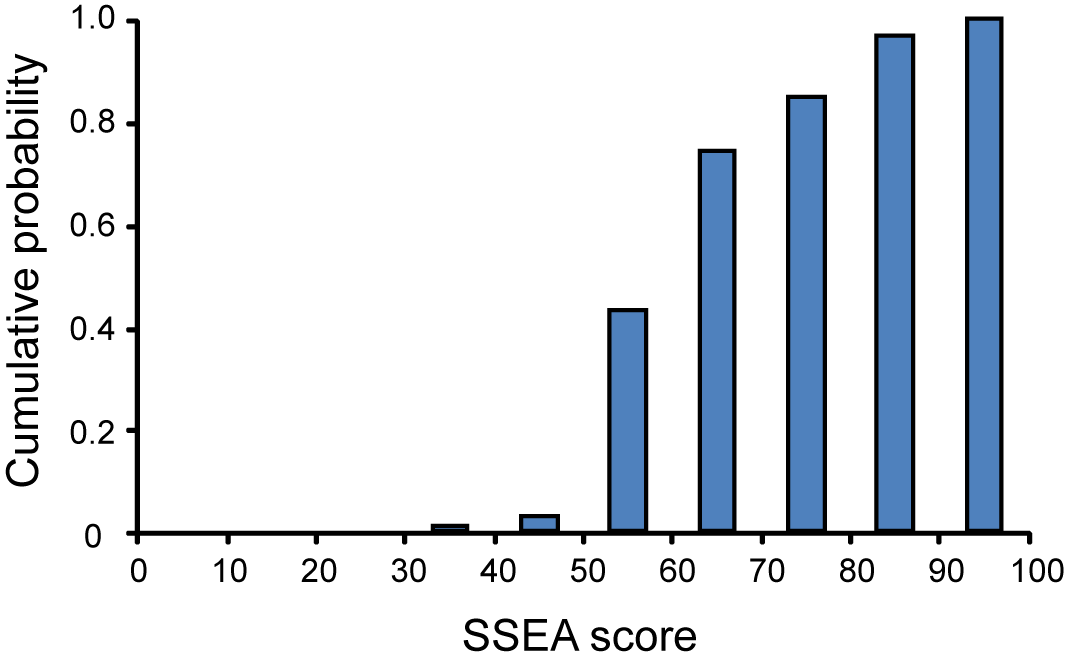

Supplement: Figure S11 — Probability of finding structure pairs with RMSD <5Å and aligned length >100 residues by SSEA scores. (TIF) [file pcbi.1002997.s011.tif]

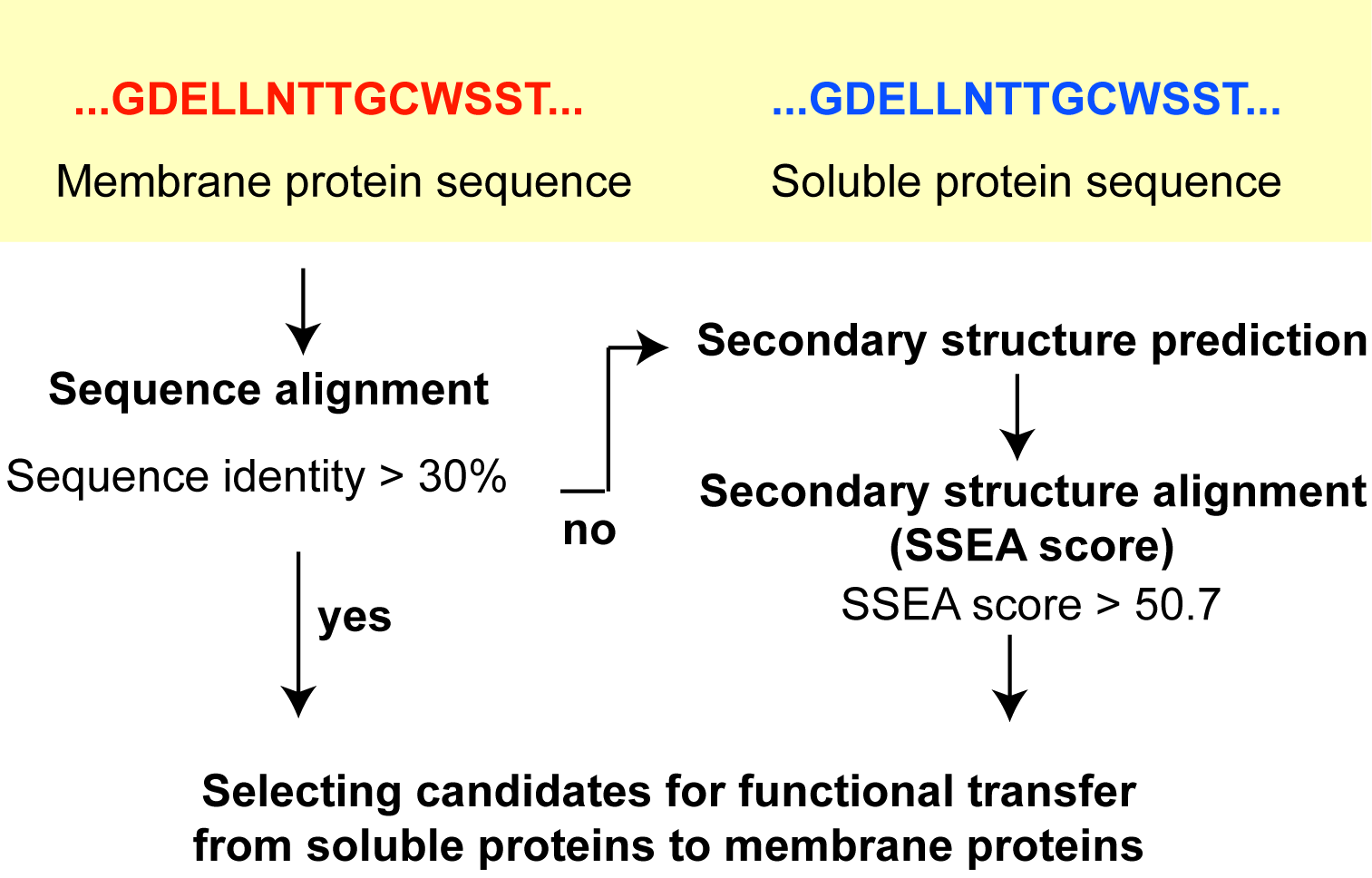

Supplement: Figure S12 — Procedure for structure-guided sequence alignment. Secondary structure element alignment was applied to select structurally comparable sequences of membrane and soluble proteins. (TIF) [file pcbi.1002997.s012.tif]

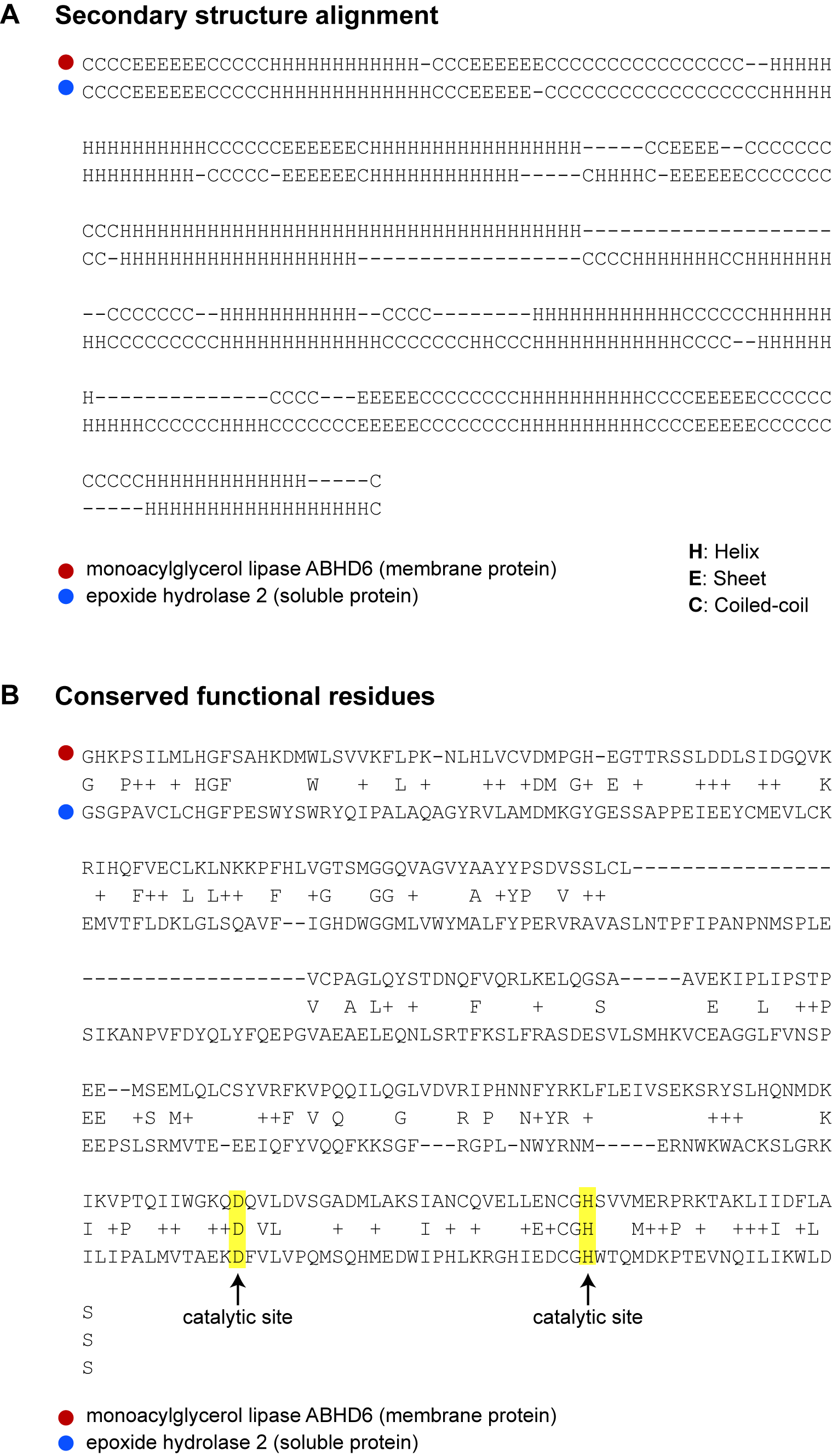

Supplement: Figure S13 — Alignment of secondary structure elements and functional residues between monoacylglycerol lipase ABHD6 and epoxide hydrolase 2. (A) Secondary structure comparison between monoacylglycerol lipase ABHD6 (membrane protein) and epoxide hydrolase 2 (soluble protein). (B) Conserved functional residues of epoxide hydrolase 2 and monoacylglycerol lipase ABHD6 were highlighted (yellow box). (TIF) [file pcbi.1002997.s013.tif]

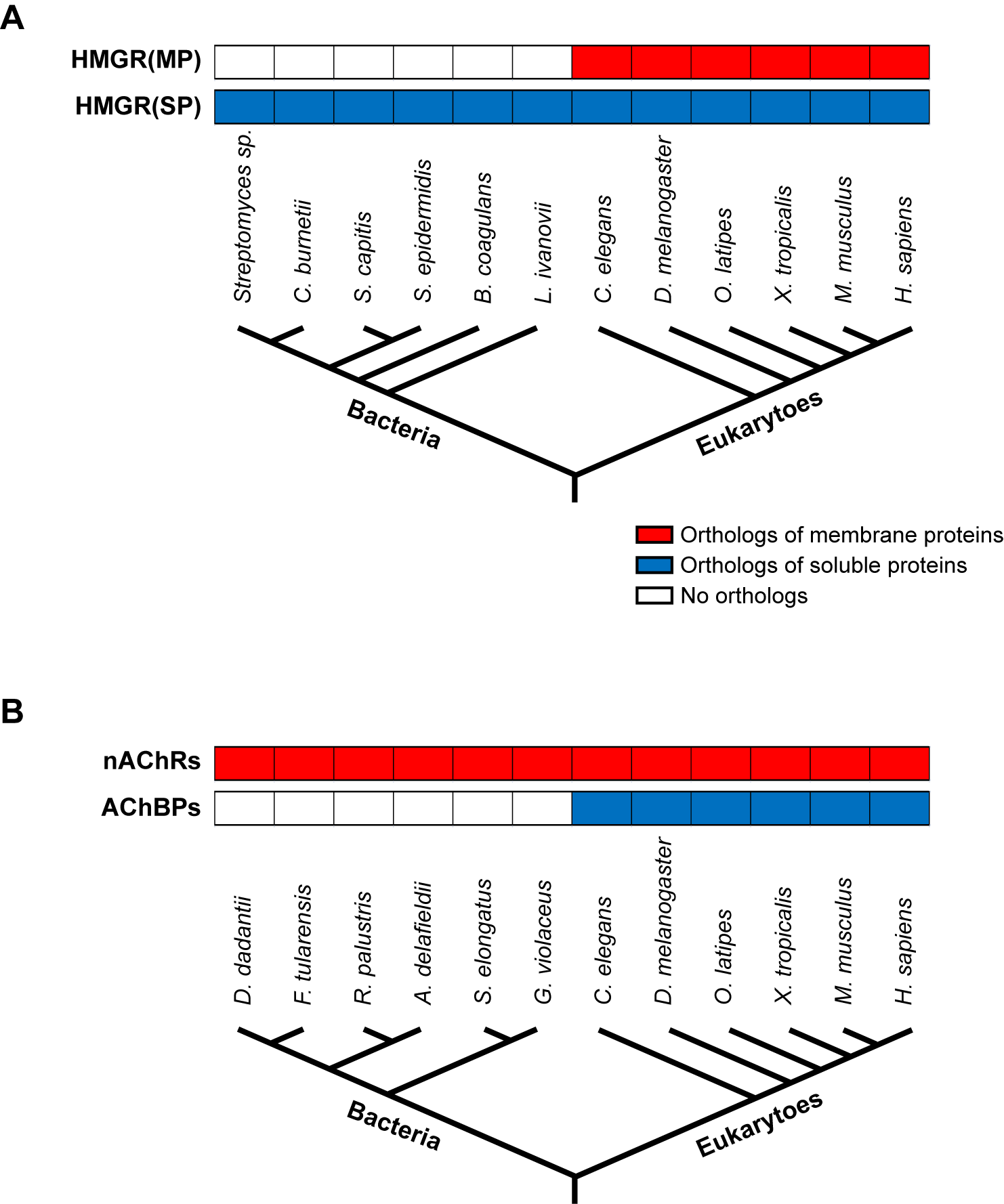

Supplement: Figure S14 — Phylogenetic profiles of membrane and soluble proteins that share extramembrane domains. (A) Phylogenetic profiles of the membrane and soluble forms of 3-hydroxy-3-methylglutaryl-CoA HMG-CoA reductase. (B) Phylogenetic profiles of nicotinic acetylcholine receptor and acetylcholine-binding protein. (TIF) [file pcbi.1002997.s014.tif]

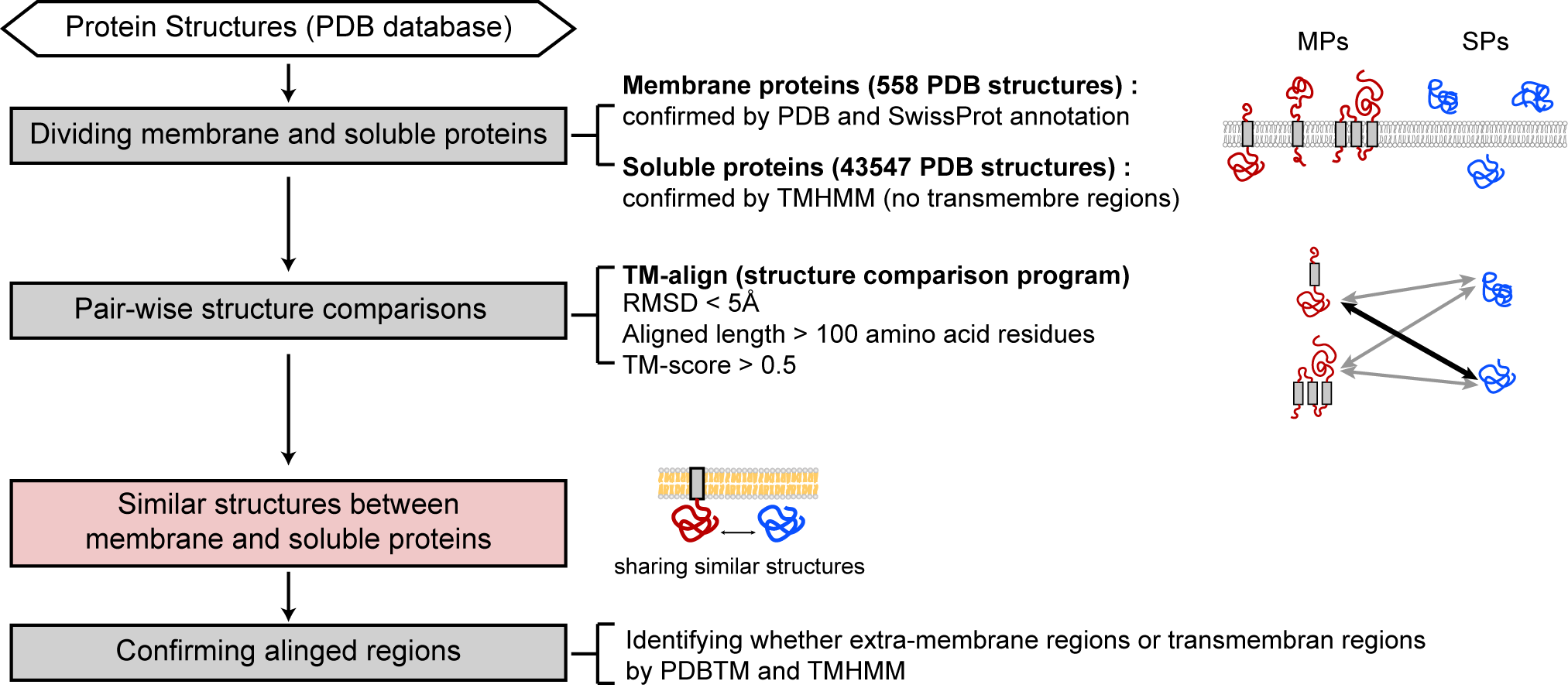

Supplement: Figure S15 — Procedure for the structure alignment of membrane and soluble proteins. (TIF) [file pcbi.1002997.s015.tif]

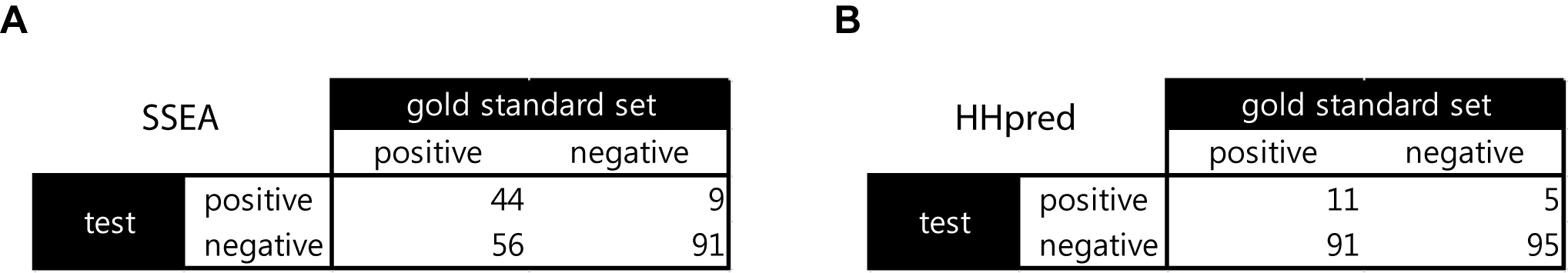

Supplement: Figure S16 — Comparison of structure-guided sequence alignment results by SSEA and HHpred. (A) Prediction results of SSEA score. (B) Prediction results of HHpred. (TIF) [file pcbi.1002997.s016.tif]
