# Supplementary material for: Rampant Exchange of the Structure and Function of Extramembrane Domains between Membrane and Water Soluble Proteins
Source: PLoS Comput Biol. 2013 Mar 21;9(3):e1002997. doi: 10.1371/journal.pcbi.1002997 (PMC3605051; doi:10.1371/journal.pcbi.1002997)
Supplement: Table S1 — Fold types of similar structure pairs between membrane and soluble proteins. (DOC) [file pcbi.1002997.s017.doc]

| Table S1. Fold types of similar structure pairs between membrane and soluble proteins | | | |
| --- | --- | --- | --- |
| **Fold name** | **MPs** | **SPs** | **Aligned pairs** |
| Periplasmic binding protein-like II | 114 | 288 | 27196 |
| Immunoglobulin-like beta-sandwich | 114 | 1003 | 6218 |
| NAD(P)-binding Rossmann-fold domains | 21 | 681 | 4344 |
| TIM beta/alpha-barrel | 20 | 1412 | 3961 |
| Streptavidin-like | 60 | 136 | 3100 |
| Flavodoxin-like | 46 | 360 | 3090 |
| P-loop containing nucleoside triphosphate hydrolases | 38 | 578 | 2575 |
| alpha/beta-Hydrolases | 9 | 392 | 1901 |
| Lipocalins | 68 | 228 | 1784 |
| Globin-like | 32 | 576 | 1558 |
| PLP-dependent transferase-like | 6 | 323 | 1004 |
| DNA/RNA-binding 3-helical bundle | 179 | 93 | 944 |
| UDP-Glycosyltransferase/glycogen phosphorylase | 10 | 188 | 938 |
| Nuclear receptor ligand-binding domain | 3 | 291 | 873 |
| Subtilisin-like | 7 | 145 | 850 |
| S-adenosyl-L-methionine-dependent methyltransferases | 13 | 257 | 674 |
| Four-helical up-and-down bundle | 49 | 117 | 629 |
| HAD-like | 14 | 134 | 614 |
| Concanavalin A-like lectins/glucanases | 10 | 335 | 609 |
| EF Hand-like | 40 | 90 | 606 |
| Periplasmic binding protein-like I | 99 | 54 | 595 |
| Ferredoxin-like | 27 | 50 | 587 |
| Ferritin-like | 44 | 109 | 559 |
| beta-lactamase/transpeptidase-like | 4 | 204 | 549 |
| lambda repressor-like DNA-binding domains | 25 | 30 | 499 |
| Glycosyl hydrolase domain | 11 | 163 | 498 |
| C-type lectin-like | 6 | 101 | 464 |
| FAD/NAD(P)-binding domain | 6 | 183 | 421 |
| Ribokinase-like | 13 | 64 | 364 |
| beta-Trefoil | 12 | 107 | 314 |
| 312 other folds |  |  |  |
