# Supplementary material for: Rampant Exchange of the Structure and Function of Extramembrane Domains between Membrane and Water Soluble Proteins
Source: PLoS Comput Biol. 2013 Mar 21;9(3):e1002997. doi: 10.1371/journal.pcbi.1002997 (PMC3605051; doi:10.1371/journal.pcbi.1002997)
Supplement: Table S2 — GO enrichment of membrane proteins that have not exchanged domains with soluble proteins. (DOC) [file pcbi.1002997.s018.doc]

| Table S2. GO enrichment of membrane proteins that have not exchanged domains with soluble proteins | |
| --- | --- |
| GO Term | P-Value |
| GO:0007186~G-protein coupled receptor protein signaling pathway | 6.78E-54 |
| GO:0007606~sensory perception of chemical stimulus | 3.15E-49 |
| GO:0007608~sensory perception of smell | 6.58E-48 |
| GO:0007600~sensory perception | 4.32E-37 |
| GO:0050890~cognition | 1.39E-31 |
| GO:0003008~system process | 6.20E-30 |
| GO:0050877~neurological system process | 7.82E-30 |
| GO:0007165~signal transduction | 4.75E-15 |
| GO:0007166~cell surface receptor linked signal transduction | 3.44E-12 |
| GO:0006811~ion transport | 3.06E-10 |
| GO:0006810~transport | 3.51E-10 |
| GO:0051234~establishment of localization | 4.50E-10 |
| GO:0050896~response to stimulus | 6.26E-07 |
| GO:0006812~cation transport | 1.89E-06 |
| GO:0015849~organic acid transport | 2.13E-06 |
| GO:0046942~carboxylic acid transport | 2.72E-06 |
| GO:0009259~ribonucleotide metabolic process | 4.05E-06 |
| GO:0051179~localization | 4.27E-06 |
| GO:0009150~purine ribonucleotide metabolic process | 5.56E-06 |
| GO:0015837~amine transport | 6.15E-06 |
