# Supplementary material for: Rampant Exchange of the Structure and Function of Extramembrane Domains between Membrane and Water Soluble Proteins
Source: PLoS Comput Biol. 2013 Mar 21;9(3):e1002997. doi: 10.1371/journal.pcbi.1002997 (PMC3605051; doi:10.1371/journal.pcbi.1002997)
Supplement: Table S5 — Common SCOP folds shared by membrane and soluble proteins. (DOC) [file pcbi.1002997.s021.doc]

| Table S5. Common SCOP folds shared by membrane and soluble proteins | |
| --- | --- |
| SCOP ID | SCOP fold name |
| 53849 | Periplasmic binding protein-like II |
| 51350 | TIM beta/alpha-barrel |
| 48725 | Immunoglobulin-like beta-sandwich |
| 51734 | NAD(P)-binding Rossmann-fold domains |
| 48507 | Nuclear receptor ligand-binding domain |
| 56435 | C-type lectin-like |
| 52171 | Flavodoxin-like |
| 49898 | Concanavalin A-like lectins/glucanases |
| 56600 | beta-lactamase/transpeptidase-like |
| 47615 | GST C-terminal domain-like |
| 52539 | P-loop containing nucleoside triphosphate hydrolases |
| 47472 | EF Hand-like |
| 54861 | Ferredoxin-like |
| 56783 | HAD-like |
| 51125 | Single-stranded right-handed beta-helix |
| 52832 | Thioredoxin fold |
| 47873 | Annexin |
| 51904 | FAD/NAD(P)-binding domain |
| 47161 | Four-helical up-and-down bundle |
| 51181 | Double-stranded beta-helix |
| 52439 | PreATP-grasp domain |
| 53821 | Periplasmic binding protein-like I |
| 55845 | N-acetylmuramoyl-L-alanine amidase-like |
| 51091 | beta-Prism I |
| 49841 | TNF-like |
| 47026 | Acyl-CoA binding protein-like |
| 46965 | Spectrin repeat-like |
| 53299 | vWA-like |
| 48064 | DBL homology domain (DH-domain) |
| 48370 | alpha-alpha superhelix |
| 50997 | 8-bladed beta-propeller |
| 55769 | Profilin-like |
| 47654 | STAT-like |
| 141657 | Pseudo beta-prism |
| 64075 | MTH938-like |
| 55297 | Bacillus chorismate mutase-like |
| 58086 | Antiparallel coiled-coil |
| 111368 | HlyD-like secretion proteins |
| 140590 | Type III secretion system domain |
