# Supplementary material for: Rampant Exchange of the Structure and Function of Extramembrane Domains between Membrane and Water Soluble Proteins
Source: PLoS Comput Biol. 2013 Mar 21;9(3):e1002997. doi: 10.1371/journal.pcbi.1002997 (PMC3605051; doi:10.1371/journal.pcbi.1002997)
Supplement: Table S6 — SwissProt domains of membrane proteins that share sequence similarity with soluble proteins. (DOC) [file pcbi.1002997.s022.doc]

| Table S6. Swiss Prot domains of membrane proteins that share sequence similarity with soluble proteins | |
| --- | --- |
| **SwissProt Domain** | **Swiss-Prot IDs of Membrane proteins** |
| Sema | RON_HUMAN |
| K_tetra | KCNV2_HUMAN |
| SRCR | C163A_HUMAN,C163B_HUMAN,ENTK_HUMAN,MSRE_HUMAN |
| Laminin_N | USH2A_HUMAN |
| ANF_receptor | GUC2D_HUMAN,GUC2C_HUMAN,NMDE2_HUMAN,GUC2F_HUMAN |
| TPR_10 | TMTC1_HUMAN |
| IL6Ra-bind | IL31R_HUMAN,IL6RA_HUMAN |
| TIG | RON_HUMAN,PLXA2_HUMAN,PLXA4_HUMAN,PLXA1_HUMAN,PLXA3_HUMAN,PLXB1_HUMAN |
| EGF | NOTC3_HUMAN,CRUM1_HUMAN,NOTC1_HUMAN,NOTC2_HUMAN,CRUM2_HUMAN,JAG2_HUMAN,DLL1_HUMAN,DLK1_HUMAN,SLIT2_HUMAN |
| Hydrolase | AT12A_HUMAN |
| TPR_11 | TOM34_HUMAN |
| Ank_5 | NOTC4_HUMAN |
| Ank_2 | ANKAR_HUMAN,NOTC3_HUMAN,TRPV5_HUMAN,TRPV2_HUMAN,TRPV6_HUMAN |
| LRRCT | TPBG_HUMAN |
| LAP2alpha | LAP2A_HUMAN |
| Ldl_recept_b | LRP2_HUMAN,EGF_HUMAN,LRP8_HUMAN,LRP1_HUMAN |
| Sel1 | SE1L2_HUMAN,SE1L1_HUMAN |
| DDHD | PITM2_HUMAN |
| Ephrin_lbd | EPHB3_HUMAN |
| PRY | ERMAP_HUMAN,BT2A3_HUMAN |
| EF_assoc_2 | MIRO2_HUMAN |
| EF_assoc_1 | MIRO2_HUMAN |
| HRM | GP124_HUMAN |
| APC_crr | APC2_HUMAN |
| CRAL_TRIO_N | S14L4_HUMAN |
| EHN | HYEP_HUMAN |
| FERM_N | FRMD3_HUMAN |
| GPS | GP126_HUMAN |
| Peptidase_M28 | NALDL_HUMAN,NALD2_HUMAN,FOLH1_HUMAN,TFR2_HUMAN |
| Kelch_4 | MEGF8_HUMAN |
| Somatomedin_B | ENPP1_HUMAN |
| cNMP_binding | HCN2_HUMAN |
| Collagen | C1QT5_HUMAN,MSRE_HUMAN,CONA1_HUMAN,COPA1_HUMAN,CODA1_HUMAN,COL12_HUMAN |
| Pep_M12B_propep | ADA21_HUMAN,ADA20_HUMAN,ADA15_HUMAN,ADA10_HUMAN,ADA28_HUMAN |
| BRCT | RFC1_HUMAN |
| CAF1 | PNDC1_HUMAN |
| I-set | UNC5B_HUMAN,LRIT2_HUMAN,ROR1_HUMAN,TUTLA_HUMAN,ROBO4_HUMAN,LRIT1_HUMAN,LRIT3_HUMAN,LRIG2_HUMAN,LRC24_HUMAN,FGRL1_HUMAN,SDK1_HUMAN,CNTN2_HUMAN,SIG16_HUMAN,LIGO3_HUMAN,CHL1_HUMAN,DSCAM_HUMAN,DSCL1_HUMAN,VGFR1_HUMAN,VGFR2_HUMAN,CEAM5_HUMAN,CDON_HUMAN |
| Ras | MIRO2_HUMAN |
| FAD_binding_8 | DUOX1_HUMAN |
| Cation_ATPase_N | AT12A_HUMAN,AT1A4_HUMAN |
| MAM | MEP1B_HUMAN |
| Arm | ANKAR_HUMAN,IMA4_HUMAN,IMA8_HUMAN,IMA7_HUMAN,IMA5_HUMAN,IMB1_HUMAN,PKP2_HUMAN |
| Thioredoxin | QSOX2_HUMAN |
| CarboxypepD_reg | CBPD_HUMAN |
| Sushi | CR2_HUMAN,SE6L2_HUMAN,CO7_HUMAN,SUSD1_HUMAN,CR1_HUMAN,SEZ6_HUMAN,LYAM2_HUMAN,LYAM3_HUMAN,CSMD2_HUMAN,MCP_HUMAN,CSMD1_HUMAN |
| A2M_N | CO5_HUMAN |
| Astacin | MEP1A_HUMAN |
| Rib_recp_KP_reg | RRBP1_HUMAN |
| zf-GRF | YD002_HUMAN |
| DUF3497 | CELR3_HUMAN,BAI1_HUMAN |
| DMAP_binding | GNPTA_HUMAN |
| Cadherin | FAT3_HUMAN,CELR2_HUMAN,FAT4_HUMAN,FAT1_HUMAN |
| zf-CCCH | U2AF4_HUMAN |
| Miro | MIRO2_HUMAN |
| fn3 | EPHB6_HUMAN,SDK1_HUMAN,USH2A_HUMAN,PTPRJ_HUMAN,INSRR_HUMAN,CDON_HUMAN,EPHA7_HUMAN,PTPRS_HUMAN,LRIT1_HUMAN,EPOR_HUMAN,TYRO3_HUMAN,UFO_HUMAN,EPHA3_HUMAN,INSR_HUMAN,NEO1_HUMAN,PTPRF_HUMAN,IL31R_HUMAN,PTPRH_HUMAN,EPHAA_HUMAN,ROS_HUMAN,TUTLA_HUMAN,NFASC_HUMAN,IL6RB_HUMAN,SDK2_HUMAN,PTPRB_HUMAN,DSCAM_HUMAN,NRCAM_HUMAN,PTPRQ_HUMAN |
| Anth_Ig | ANTRL_HUMAN |
| GDPD | GDE1_HUMAN,GDPD1_HUMAN |
| PX | NOXO1_HUMAN |
| CSF-1 | CSF1_HUMAN |
| Methyltransf_3 | COMT_HUMAN |
| Pkinase_Tyr | STYK1_HUMAN,DDR2_HUMAN,EPHA6_HUMAN,LMTK1_HUMAN,UFO_HUMAN,GUC2D_HUMAN,PGFRB_HUMAN,EPHA8_HUMAN |
| PA | TFR2_HUMAN |
| PG_binding_1 | MMP25_HUMAN |
| SNARE | STX4_HUMAN |
| Alpha-amylase | 4F2_HUMAN |
| LBP_BPI_CETP_C | BPI_HUMAN |
| FKBP_C | FKB11_HUMAN |
| RRM_1 | U2AF4_HUMAN,NUCL_HUMAN |
| Amidase | FAAH2_HUMAN,FAAH1_HUMAN |
| C2-set_2 | BT1A1_HUMAN,SIG10_HUMAN,SN_HUMAN |
| Mito_carr | SCMC3_HUMAN |
| Kelch_5 | ATRN1_HUMAN |
| F5_F8_type_C | DCBD1_HUMAN |
| SH3_1 | NOXA1_HUMAN,NOXO1_HUMAN |
| Glyco_hydro_1 | KLOT_HUMAN,LPH_HUMAN,KLOTB_HUMAN |
| NIDO | NID2_HUMAN |
| Alpha-mann_mid | MA2A1_HUMAN |
| DSL | DLL4_HUMAN |
| Macscav_rec | MSRE_HUMAN |
| Cu2_monooxygen | AMD_HUMAN |
| Disintegrin | ADA21_HUMAN,ADA20_HUMAN,ADAM8_HUMAN,ADA29_HUMAN |
| Acetyltransf_1 | SAT2_HUMAN,NAT8_HUMAN |
| Pkinase | ERN1_HUMAN,TAOK2_HUMAN,AMHR2_HUMAN |
| DUF1053 | ADCY7_HUMAN |
| 3Beta_HSD | 3BHS1_HUMAN |
| PAS_9 | KCNH4_HUMAN |
| PSI | PLXA3_HUMAN,PLXA4_HUMAN,SEM5A_HUMAN,SEM6D_HUMAN,PLXA1_HUMAN |
| Furin-like | INSRR_HUMAN |
| Filamin | FLNB_HUMAN |
| Crystall | AIM1_HUMAN |
| Melibiase | AGAL_HUMAN |
| IR1-M | RBP2_HUMAN |
| Ig_3 | NRCAM_HUMAN,LIRA1_HUMAN,LIRA5_HUMAN,LIRA4_HUMAN,LIRA6_HUMAN,CEAM8_HUMAN,FCRL3_HUMAN,CNTN2_HUMAN,CEAM5_HUMAN |
| Ig_2 | FCGRB_HUMAN,SDK1_HUMAN,TIE1_HUMAN,GPA33_HUMAN,FCRL3_HUMAN,GPVI_HUMAN,CEAM5_HUMAN,CDON_HUMAN,NTRK1_HUMAN,FCG2A_HUMAN,LIRA5_HUMAN,LIRA4_HUMAN,CEAM6_HUMAN,FCGR1_HUMAN,UFO_HUMAN,SIG14_HUMAN,SIG10_HUMAN,DSCL1_HUMAN,ICAM5_HUMAN,SIGL5_HUMAN,OSCAR_HUMAN,PSG8_HUMAN,BOC_HUMAN,VSTM1_HUMAN,CEA21_HUMAN,MUSK_HUMAN,PSG7_HUMAN,PSG1_HUMAN,FCERA_HUMAN,FCRL5_HUMAN,TUTLA_HUMAN,FCGRC_HUMAN,NFASC_HUMAN,PVRL2_HUMAN,LIRA1_HUMAN,IGSF1_HUMAN,FCRL4_HUMAN,SDK2_HUMAN,VGFR1_HUMAN,AMGO2_HUMAN,CHL1_HUMAN,CEAM7_HUMAN,SIG16_HUMAN,SN_HUMAN |
| ANATO | CO5_HUMAN |
| Abhydrolase_5 | ABHEA_HUMAN |
| zf-RanBP | RBP2_HUMAN |
| GOLD_2 | S14L4_HUMAN |
| Laminin_G_2 | CNTP4_HUMAN,CNTP1_HUMAN,CNTP5_HUMAN,USH2A_HUMAN,CNTP3_HUMAN,CRUM2_HUMAN,SLIT2_HUMAN |
| Laminin_G_3 | USH2A_HUMAN |
| MATH | MEP1B_HUMAN |
| Abhydrolase_3 | ADCL3_HUMAN,AAAD_HUMAN,ADCL4_HUMAN |
| Thyroglobulin_1 | NID2_HUMAN |
| Ldl_recept_a | LRP1B_HUMAN,ENTK_HUMAN,RXFP2_HUMAN,TMPS9_HUMAN,VLDLR_HUMAN,LRP2_HUMAN,TMPS6_HUMAN,CORIN_HUMAN,ST14_HUMAN |
| Laminin_EGF | SREC_HUMAN,USH2A_HUMAN,MEG11_HUMAN,MEG10_HUMAN,PEAR1_HUMAN,MEGF9_HUMAN |
| Cu_amine_oxid | AOC3_HUMAN |
| LRRNT | GP1BA_HUMAN,VASN_HUMAN,LRC52_HUMAN,LRTM2_HUMAN,LIGO3_HUMAN |
| Glyco_trans_4_4 | ALG2_HUMAN |
| Ant_C | ANTR2_HUMAN |
| MNNL | DLL3_HUMAN |
| Trypsin | TMPSC_HUMAN,TRYG1_HUMAN,ENTK_HUMAN,TM11F_HUMAN,TM11B_HUMAN,TM11A_HUMAN,TMPS5_HUMAN,TMPS9_HUMAN |
| Hemopexin | MMP25_HUMAN,MMP16_HUMAN,MMP17_HUMAN |
| Kunitz_BPTI | SPIT2_HUMAN |
| zf-H2C2_2 | ZNF32_HUMAN |
| GSHPx | GPX8_HUMAN |
| TNFR_c6 | TNR1A_HUMAN |
| Fz | MUSK_HUMAN |
| V-set | CEA16_HUMAN,VSIG8_HUMAN,GPA33_HUMAN,CEAM5_HUMAN,AMGO1_HUMAN,IGS11_HUMAN,UFO_HUMAN,BTNL2_HUMAN,CD8A_HUMAN,TPSNR_HUMAN,SLAF6_HUMAN,VSIG2_HUMAN,CD80_HUMAN,SIG12_HUMAN,CEA21_HUMAN,PSG7_HUMAN,IGSF3_HUMAN,BTNL9_HUMAN,VGFR2_HUMAN,TVA2_HUMAN,PSG1_HUMAN,PIGR_HUMAN |
| Glyco_hydro_56 | HYAL4_HUMAN |
| TFR_dimer | NALD2_HUMAN,TFR2_HUMAN |
| SEA | TM11F_HUMAN,TM11B_HUMAN,ST14_HUMAN,ENTK_HUMAN |
| adh_short | DHB7_HUMAN,RDH8_HUMAN,SPRE_HUMAN,RDH16_HUMAN,KDSR_HUMAN |
| VWC | FRAS1_HUMAN |
| C2 | SYT13_HUMAN,FR1L6_HUMAN |
| FMO-like | FMO6_HUMAN |
| FerA | DYSF_HUMAN |
| C1-set | IGHM_HUMAN |
| Glyco_transf_7N | B4GT2_HUMAN |
| FerB | FR1L6_HUMAN |
| PDZ | DLG3_HUMAN |
| ABC_tran | MRP9_HUMAN,ABCA7_HUMAN,ABCCB_HUMAN,ABCA9_HUMAN,ABCAD_HUMAN,ABCA8_HUMAN,MRP6_HUMAN,ABCBA_HUMAN,MRP3_HUMAN,ABCB5_HUMAN,ABCAA_HUMAN |
| cEGF | EMR1_HUMAN,C1QR1_HUMAN,LRP1_HUMAN |
| Cu_amine_oxidN2 | AOC3_HUMAN |
| Glyco_transf_7C | GLT11_HUMAN |
| LRR_8 | LRC4B_HUMAN,LRC59_HUMAN,LRFN3_HUMAN,TPBG_HUMAN,LRIT2_HUMAN,LRIT1_HUMAN,LRIT3_HUMAN,FLRT3_HUMAN,LIGO2_HUMAN,FSHR_HUMAN,LRC66_HUMAN,LRC33_HUMAN,LRC55_HUMAN,SLIK6_HUMAN,LRRN1_HUMAN,LRRN2_HUMAN,LRC26_HUMAN,GP124_HUMAN,LRC15_HUMAN,VASN_HUMAN,GPV_HUMAN,FLRT1_HUMAN,LRTM1_HUMAN,LRC32_HUMAN,LRC24_HUMAN,LRIG1_HUMAN,LIGO3_HUMAN |
| TPR_1 | TMTC1_HUMAN,TOM34_HUMAN,RGPD8_HUMAN |
| CUB | ENTK_HUMAN,MFRP_HUMAN,CUZD1_HUMAN,DCBD1_HUMAN,CSMD2_HUMAN,ST14_HUMAN |
| Kv2channel | KCNB2_HUMAN |
| His_Phos_2 | PPAT_HUMAN |
| LRR_1 | LRRC4_HUMAN,LRRT1_HUMAN,LRC32_HUMAN,SLIK3_HUMAN,LRC33_HUMAN,LRC52_HUMAN,LGR5_HUMAN,FLRT1_HUMAN,SLIK1_HUMAN,TLR7_HUMAN,TLR8_HUMAN,TLR5_HUMAN,LRC8D_HUMAN,TLR3_HUMAN,LRC4C_HUMAN,LGR6_HUMAN,AMGO1_HUMAN,SLIT2_HUMAN,LRC38_HUMAN |
| LRR_4 | LGR5_HUMAN,LRC8E_HUMAN,LRIG3_HUMAN |
| LRR_5 | LRC8A_HUMAN,LRIG2_HUMAN |
| LRR_6 | LRC38_HUMAN,LRFN1_HUMAN,LRRT2_HUMAN |
| LRR_7 | LGR5_HUMAN,LRC33_HUMAN,GPV_HUMAN,TLR9_HUMAN |
| WIF | RYK_HUMAN |
| Metallophos | PPA5_HUMAN |
| SPRY | BT2A3_HUMAN,BTNL3_HUMAN |
| ZU5 | UNC5B_HUMAN |
| NTR | CO5_HUMAN |
| Ank | TNKS1_HUMAN,TRPA1_HUMAN,NOTC1_HUMAN,ZDH17_HUMAN |
| NOD | NOTC3_HUMAN,NOTC1_HUMAN |
| Alk_phosphatase | PPBI_HUMAN |
| Aminotran_1_2 | SPTC3_HUMAN,ALAT1_HUMAN |
| Integrin_B_tail | ITB5_HUMAN |
| Ephrin | EFNA1_HUMAN,EFNA4_HUMAN |
| AAA | AFG32_HUMAN,RFC1_HUMAN |
| IBB | IMA8_HUMAN |
| CAP | GLIP1_HUMAN,PI16_HUMAN,GRPL2_HUMAN |
| NTF2 | NXF1_HUMAN |
| Cu-oxidase_3 | HEPH_HUMAN |
| Lectin_C | CLC4D_HUMAN,KLRG1_HUMAN,CLC4K_HUMAN,PLA2R_HUMAN,PRG3_HUMAN,ASGR1_HUMAN,CL12B_HUMAN,CLC10_HUMAN,CLC4E_HUMAN,COL12_HUMAN,PK1L2_HUMAN,CLC6A_HUMAN,CLC9A_HUMAN,CLC2A_HUMAN,CLC5A_HUMAN,CLC2B_HUMAN,MRC2_HUMAN,CLC1B_HUMAN,CLC4C_HUMAN,MRC1_HUMAN,CLC2D_HUMAN,CLC4A_HUMAN,LY75_HUMAN,FCER2_HUMAN |
| hEGF | SREC_HUMAN,PEAR1_HUMAN,DNER_HUMAN,CRUM2_HUMAN,JAG2_HUMAN,DLK2_HUMAN,DLL4_HUMAN |
| Phosphodiest | ENPP7_HUMAN,ENPP3_HUMAN,ENPP4_HUMAN |
| Ricin_B_lectin | GALT8_HUMAN |
| ig | CD22_HUMAN,SIGL5_HUMAN,KI2S5_HUMAN,FCRL4_HUMAN,FCGR1_HUMAN,NTRK3_HUMAN,PGFRB_HUMAN,IGHD_HUMAN |
| ADAM_CR | ADAM8_HUMAN,ADA20_HUMAN,ADAM2_HUMAN,ADAM7_HUMAN,ADA12_HUMAN,ADA29_HUMAN |
| GBP_C | GBP7_HUMAN |
| Acyltransferase | PCAT2_HUMAN |
| NHL | AMD_HUMAN |
| EpoR_lig-bind | EPOR_HUMAN |
| Lipocalin | CO8G_HUMAN |
| Gal_Lectin | PK1L2_HUMAN |
| Fasciclin | STAB2_HUMAN,STAB1_HUMAN |
| Peptidase_M10 | MMP25_HUMAN |
| Snurportin1 | SPN1_HUMAN |
| Glyco_hydro_38 | MA2A1_HUMAN,MA2A2_HUMAN |
| Calx-beta | FRAS1_HUMAN |
| Integrin_alpha2 | ITA1_HUMAN |
| Glycos_transf_2 | GALT8_HUMAN,GLTL5_HUMAN,GLT11_HUMAN,GALT7_HUMAN,GALT9_HUMAN,GLT14_HUMAN |
| p450 | CP4Z1_HUMAN,CP4V2_HUMAN,CP8B1_HUMAN |
| Transferrin | TRFM_HUMAN |
| Latexin | TIG1_HUMAN |
| GCC2_GCC3 | EPHB6_HUMAN |
| Vault | MVP_HUMAN |
| TNF | TNF10_HUMAN,TNF18_HUMAN,TNF15_HUMAN |
| Peptidase_M1 | AMPN_HUMAN |
| NODP | NOTC4_HUMAN |
| Reprolysin | ADA21_HUMAN,ADA20_HUMAN,ADEC1_HUMAN,ADA18_HUMAN,ADA11_HUMAN |
| PB1 | NOXA1_HUMAN |
| EF_hand_5 | SCMC3_HUMAN |
| Notch | NOTC3_HUMAN,NOTC1_HUMAN |
| TPR_2 | NOXA1_HUMAN,TOM34_HUMAN |
| EGF_CA | NOTC3_HUMAN,NOTC1_HUMAN,LRP1B_HUMAN,JAG1_HUMAN,JAG2_HUMAN,NOTC4_HUMAN,FAT1_HUMAN,EGF_HUMAN |
| G_glu_transpept | GGT3_HUMAN,GGT6_HUMAN |
| SAM_2 | EPHA7_HUMAN |
| PTEN_C2 | TPTE2_HUMAN,TPTE_HUMAN |
| Engrail_1_C_sig | HME1_HUMAN |
| TSP_1 | UNC5B_HUMAN,UNC5D_HUMAN,THS7A_HUMAN,THS7B_HUMAN,SEM5A_HUMAN,SEM5B_HUMAN,BAI1_HUMAN |
| VWA | ANTRL_HUMAN,ITA1_HUMAN |
| DUF3454 | NOTC3_HUMAN |
| Activin_recp | ACVL1_HUMAN |
| Glyco_hydro_38C | MA2A2_HUMAN |
| EGF_3 | STAB2_HUMAN,STAB1_HUMAN |
| SAMP | APC2_HUMAN |
| An_peroxidase | DUOX2_HUMAN |
